# Strain-specific strategies of 2′-fucosyllactose, 3-fucosyllactose, and difucosyllactose assimilation by *Bifidobacterium longum* subsp. *infantis* Bi-26 and ATCC 15697

**DOI:** 10.1038/s41598-020-72792-z

**Published:** 2020-09-28

**Authors:** Bryan E. Zabel, Svetlana Gerdes, Kara C. Evans, Derek Nedveck, Suzanne Koch Singles, Barbara Volk, Charles Budinoff

**Affiliations:** 1 Genomics and Microbiome Science, DuPont Nutrition and Biosciences, Madison, WI USA; 2 Advanced Analytical, DuPont Nutrition and Biosciences, Wilmington, DE USA

**Keywords:** Biochemistry, Cell biology, Genetics, Microbiology, Molecular biology

## Abstract

Human milk provides essential nutrients for infant nutrition. A large proportion of human milk is composed of human milk oligosaccharides (HMOs), which are resistant to digestion by the infant. Instead, HMOs act as a bioactive and prebiotic enriching HMO-utilizing bacteria and cause systematic changes in the host. Several species of *Bifidobacterium* have been shown to utilize HMOs by conserved, as well as species-specific pathways, but less work has been done to study variation within species or sub-species. *B. longum* subsp. *infantis* is a prevalent species in the breast-fed infant gut and the molecular mechanisms of HMO utilization for the type strain *B. longum* subsp. *infantis* ATCC 15697 (type strain) have been well characterized. We used growth, transcriptomic, and metabolite analysis to characterize key differences in the utilization of 2′FL, 3FL and DFL (FLs) between *B. longum* subsp. *infantis* Bi-26 (Bi-26) and the type strain. Bi-26 grows faster, produces unique metabolites, and has a distinct global gene transcription response to FLs compared to the type strain. Taken together the findings demonstrate major strain specific adaptations in Bi-26 to efficient utilization of FLs.

## Introduction

Human milk is recognized as the optimal food source for infant nutrition, providing the infant with the needed macronutrients, micronutrients and growth promoting factors needed for infant development, with its composition changing with age or needs of the infant^1,2^. A unique characteristic of human milk is the large proportion of non-digestible, human milk oligosaccharides (HMOs), with their total concentration of 5–20 g/L only exceeded by lactose and total lipids^1^. HMOs nutritional value for an infant is negligible, as intestinal human enzymes do not hydrolyze the majority of the glycosidic bonds found in these compounds, yet they act as a bioactive compound providing an array of benefits to the infant, including anti-adhesive effects against pathogens, anti-inflammatory modulation of the intestinal epithelial response, and development of the immune system. Importantly HMOs also function as prebiotics for beneficial bacteria, enriching the populations capable of efficiently utilizing these substrates in the developing infant colon, often dominated by bifidobacteria^3–6^.

Over 200 structurally distinct HMOs have been identified^7^ with chain lengths from 3 to 15 carbohydrate units, composed of five monosaccharides: glucose, galactose, *N*-acetylglucosamine, fucose, and *N*-acetylneuraminic acid (also known as sialic acid). These core oligosaccharides are often fucosylated by adding fucose (via α1–2/3/4 linkages) and/or sialylated by adding sialic acid via 2–3/6 linkages^5,8,9^. In spite of this complexity, a relatively small number of HMO species can represent up to 70% of the total in an individual mother’s milk^10^. Most women primarily produce fucosylated HMOs with 2′-fucosyllactose (2′FL), 3-fucosyllactose (3FL) and difucosyllactose (DFL or LDFT) being the simplest forms. Typically, fucosylated HMOs make up the majority (> 40%) of the total HMOs found in breast milk unless the milk is derived from non-secretor and/or Lewis negative mothers^8,9,11^. The fucosylated trisaccharide 2′-fucosyllactose (Fucα1–2Galβ1–4Glc, 2′FL) is the most abundant HMO^8,9,11^ representing from 12 to 45% of the total HMO content in individual mothers’ milk^12^, while 3-fucosyllactose (Galβ1–4(Fucα1–3)Glc, 3FL) is less abundant, representing from 0.5% to 3% of total HMOs^12^.

Bifidobacteria are Gram-positive anaerobes belonging to the Actinobacteria phylum. They are important commensals of the human gut and are enriched in the infant gut, especially in breast-fed infants^13,14^. The genus *Bifidobacterium* encompasses 70 recognized species and 10 subspecies^15^, while only a few species are capable of utilizing HMOs as their sole carbon source and are prevalent in the gut microbiota of breastfed infants^13,14^. These are typically dominated by the species *B. breve*,* B. longum* subsp. *longum* and *B. bifidum*, with *B. pseudocatenulatum*,* B. longum* subsp. *infantis*,* B. longum* subsp. *suis*, and *B. kashiwanohense* occurring at lower frequency^14,16–19^. However, there does not seem to be a strict divide between infant and adult associated bifidobacterial taxa, understandable in the light of vertical transfer of bifidobacterial species from mother to infant, which can include adult symbionts such as *B. adolescentis*^13^.

The physiology and molecular mechanisms of HMO utilization for the type strain *B. infantis* ATCC 15697 have been extensively studied. However, less research has evaluated strain-level variation in these mechanisms across this subspecies*.* In this study we used comparative genomics, metabolite analysis and transcriptomics to analyze the growth of the type strain and *B. infantis* strain Bi-26 (Bi-26) on 2′FL, 3FL, and DFL (FLs) as sole carbon sources. Our results suggest that Bi-26 and the type strain utilize different metabolic strategies during growth on FLs. Namely, the type strain is a generalist, utilizing different fractions of HMO equally, while Bi-26 is specifically adapted for fast and efficient utilization of FLs.

## Results

### Genomics overview of the *B. longum* subsp.* infantis* group

**Figure 1:**
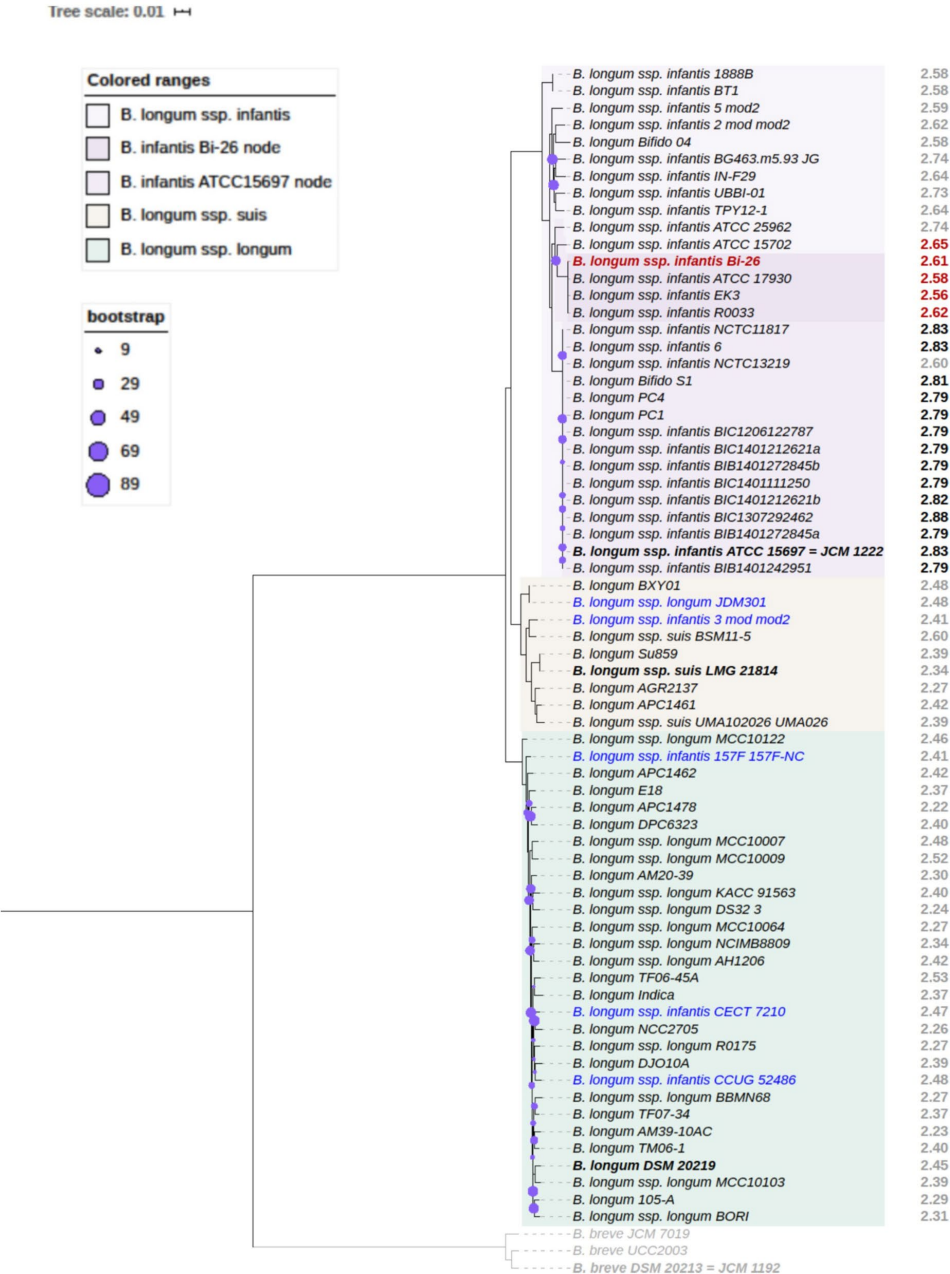
Phylogenetic tree of the *B. longum* species based on 500 core proteins using RAxML in the Phylogenetic Tree Building Service of the PATRIC database^84^. Support values were generated using 100 rounds of the ‘Rapid’ bootstrapping option of RAxML and only bootstrap values lower than 90 are shown. Tree scale is given in amino acid substitutions per site. Genome sizes (Mb) are shown to the right of each leaf: genomes larger than 2.75 Mb are in black, and genomes of 2.74 Mb or smaller and in gray, except those clustering with the Bi-26 node (red). Genomes from the subspecies *B. longum* subsp. *suis* are shown in beige and *B. longum* subsp. *infantis* in purple, with the Bi-26 clade highlighted in deeper shade. Representative genomes from the subspecies *B. longum* subsp. *longum* (turquoise) were chosen for the maximum representation of the inter-subspecies diversity from ~ 300 nonredundant genomes of this subspecies available in PATRIC as of October 2019. Several *B. breve* genomes (sister clade) included for rooting (shown in gray). Type strains are in bold. Organism names are shown as they appear in public databases; about a third of the *B. longum* species genomes are currently not typed to the subspecies level or are misidentified (shown in blue).

We identified 30 publicly available genomes that cluster with the type strain (ATCC 15697) to explore the genomic variation in *B. longum* subsp. *infantis* strains (Fig. 1). Since many *B. longum* genomes in the public databases are not typed to the subspecies or are misidentified (highlighted blue in Fig. 1), we used robust tree building to identify all *B. longum* genomes that fall into the *B. infantis* subspecies clade*.*

**Figure 2:**
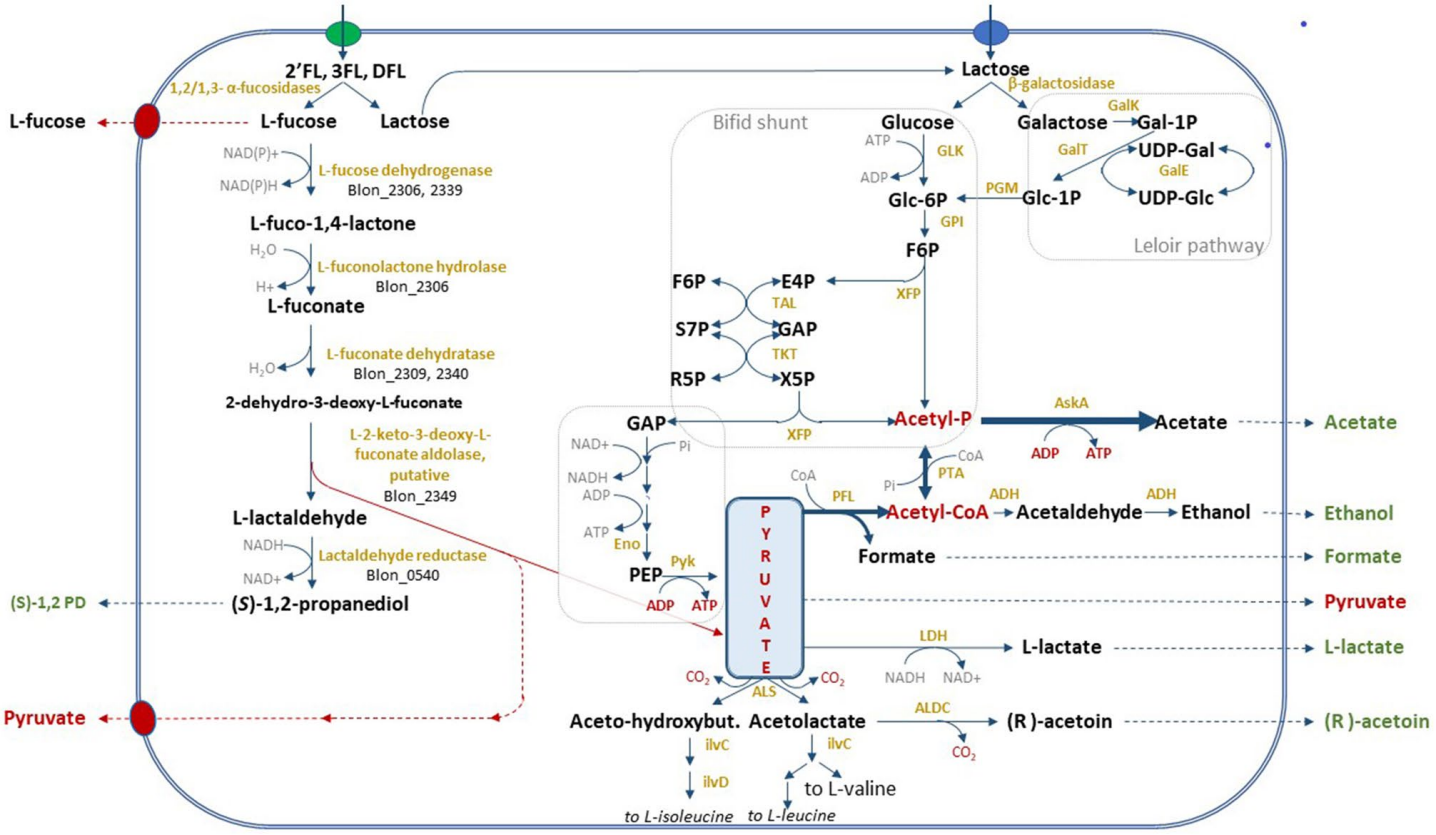
*Bifidobacterium longum* subsp. *infantis* metabolic pathway for utilization of 2′FL, 3FL, DFL and their constituent monosaccharides. FLs are internalized by ABC transporters and hydrolyzed intracellularly by a set of β-galactosidases and α-fucosidases of various substrate specificities (listed in Fig. 7). The resultant monosaccharides enter the Bifid shunt and fermentative pathways characteristic of this taxon. L-fucose catabolism in bifidobacteria has not yet been fully elucidated and is depicted as predicted in^19,23^ with modifications. Known or predicted genes implicated in L-fucose catabolism are shown as Blon IDs. The central role of the pyruvate node for metabolism of small fucosylated HMOs is highlighted. Dissipation of the excess of L-fucose via yet unidentified efflux transporters for fucose and pyruvate is shown on the left. Enzymes are in gold, compounds in black, secreted fermentation end products are in green or red (for pyruvate). See Supplemental Table 2 for explanation of abbreviations.

Our strain of interest, Bi-26, clusters in a separate sub-clade from the type strain. The genomes of Bi-26 and the type strain are composed of 2,608,123 bp and 2,832,748 bp, respectively, and contain 2411 and 2486 coding sequences (CDS), respectively. While both genomes have a similar number of coding genes, only 80% of these CDSs are orthologous (~ 1960 genes). The strain-specific genes (505 in Bi-26 and 580 in the type strain) largely fall into the following categories: EPS/capsular biosynthesis enzymes, restriction-modification systems, transporters, mobile elements, and hypotheticals. The type strain genome is 8% (224.6 kb) larger than Bi-26, with three quarters of the difference due to a large 164.7 kb genomic island (Blon_1175–Blon_1354) devoid of almost any genes with identifiable functions, except those potentially associated with prophage or mobile elements. This island has been reported before in ATCC 15697 as ‘a very large phage insert, region M-3 rich in mobile elements’^20^. This genomic island can be found in whole or in part in less than a dozen (as of April 2020) other *B. longum* genomes in public databases out of ~ 1200 total. The fact that so few genomes in the *B. longum* species carry this locus indicates that it is unessential for *B. longum* survival. The remainder of the genome size difference is due to selected gene loss in Bi-26, largely of various transporters and glycosidases, which likely reflects adaptation to utilization of specific carbohydrates (illustrated in Fig. 7). Analysis of the presence or absence of orthologous genes between the genomes of Bi-26 and the type strain showed that the complement of core metabolic enzymes involved in HMO utilization identified earlier in the type strain^20–22^ and other bifidobacterial strains and species^23–25^ is stable, while the set of HMO transporters and glycosidases varies between the two strains. The metabolic map of FL utilization by Bi-26 (Fig. 2) shows the expected metabolites produced and the genes necessary to process FLs (Supplemental Table 2). Out of 17 HMO utilization genes missing from Bi-26, 11 encode subunits of transporters of various substrate specificity and 4 pertain to glycosidases (two β-galactosidases and two α-fucosidases) (Fig. 7).

Comparison of genome sizes between the 30 *B. longum* subsp. *infantis* strains in Fig. 1 shows that the entire group of *B. infantis* genomes that fall on the same node with Bi-26 are smaller than genomes clustering with the type strain. The average genome size of the former group is 2606 kb, while the average size of the latter group is 7.3% larger: 2796 kb (Fig. 1).

Also of interest was a transposase insertion in the antiterminator required for activity of the β-glucoside-specific PTS transport system (Blon_2183) involved in recognition and uptake of glucose from the external environment^26^. This transposase is not present in Bi-26, but present in the type strain and several other strains of the subspecies.

### Growth of Bi-26 and the type strain on FLs

**Figure 3:**
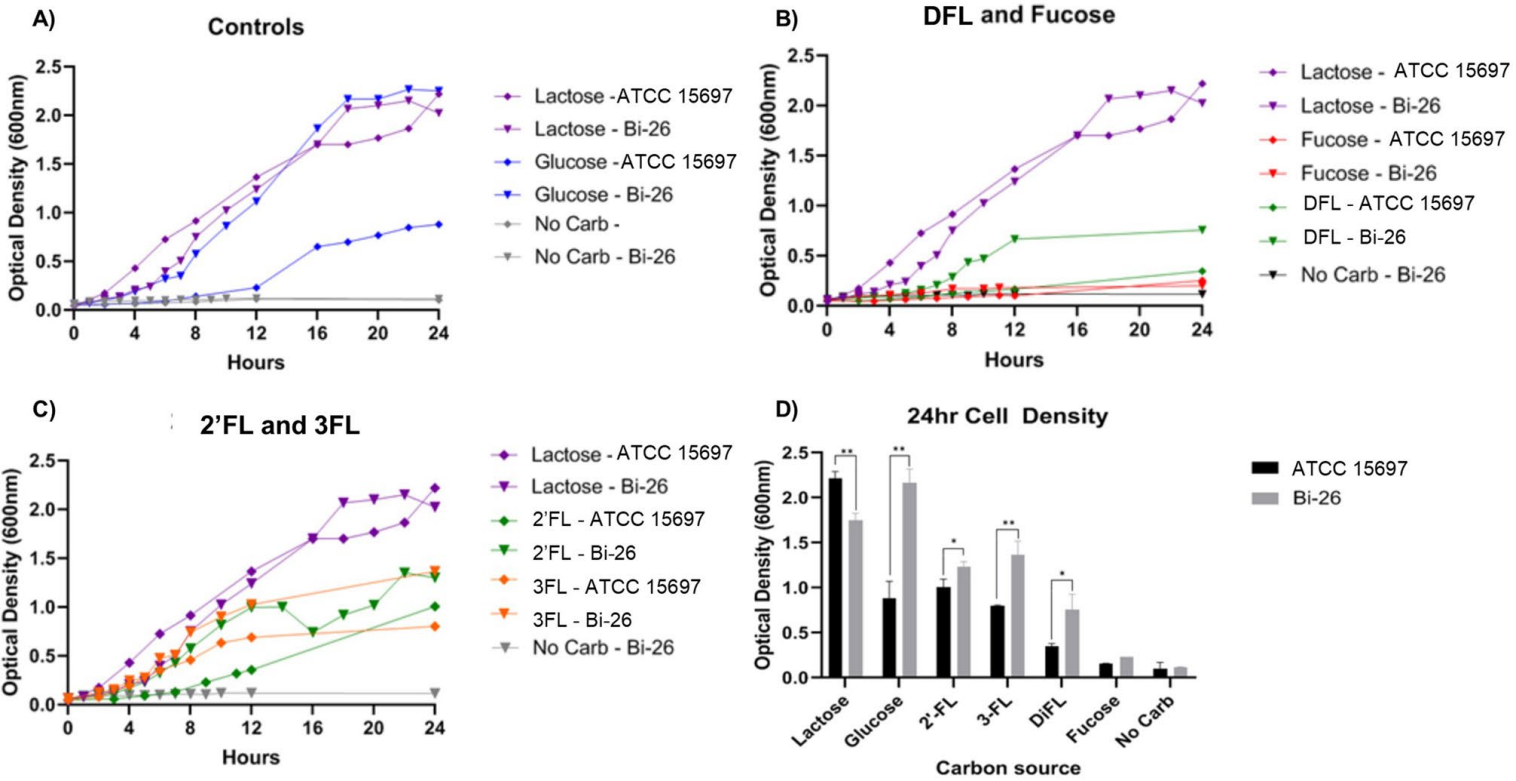
HMO growth and utilization for Bi-26 and ATCC 15697. Cell density (OD_600_) was measured every 1–2 h for 24 h with glucose, lactose, 2′FL, 3FL, DFL, or fucose as the sole carbon source (**A**–**C**). Growth curve values are an average of 3 independent experiments. Cell density measured at 24 h (**D**). Tests of difference between Bi-26 and the type strain were done using unpaired t-tests. Error bars represent 1 standard deviation from the mean (n = 3). Bi-26 had higher cell density measurements at 24 h in all conditions except fucose and lactose (however, Bi-26 cell density exceeded that of the type strain on lactose at the three earlier time points). *p value ≤ 0.05; **p value ≤ 0.005.

Growth of Bi-26 and the type strain was monitored (OD_600_) in mBasal media over 24 h. To determine potential growth differences, the strains were grown on 2′FL, 3FL, DFL, lactose, glucose or fucose as the sole carbon source. Maximum growth was seen with lactose for the type strain and glucose for Bi-26 with nearly identical final cell density measurements (Fig. 3A). While both strains grew similarly on lactose, the type strain only reached half the cell density of Bi-26 when grown on glucose (Fig. 3D).

For both strains, growth on 2′FL and 3FL resulted in a lower cell density at 24 h (Fig. 3D) compared to lactose. The type strain grew significantly slower on 2′FL compared to 3FL with only the 24-h time point results showing less growth on 3FL than on 2-FL (Fig. 3C). Bi-26 showed significantly faster growth (Fig. 3C) and higher final cell density than the type strain on 2′FL and 3FL (Fig. 3D). When comparing DFL growth the type strain had about half of the final cell density compared to the other FLs with Bi-26 producing a significantly higher cell density on this carbon source than ATCC 15697 (Fig. 3B).

**Figure 4:**
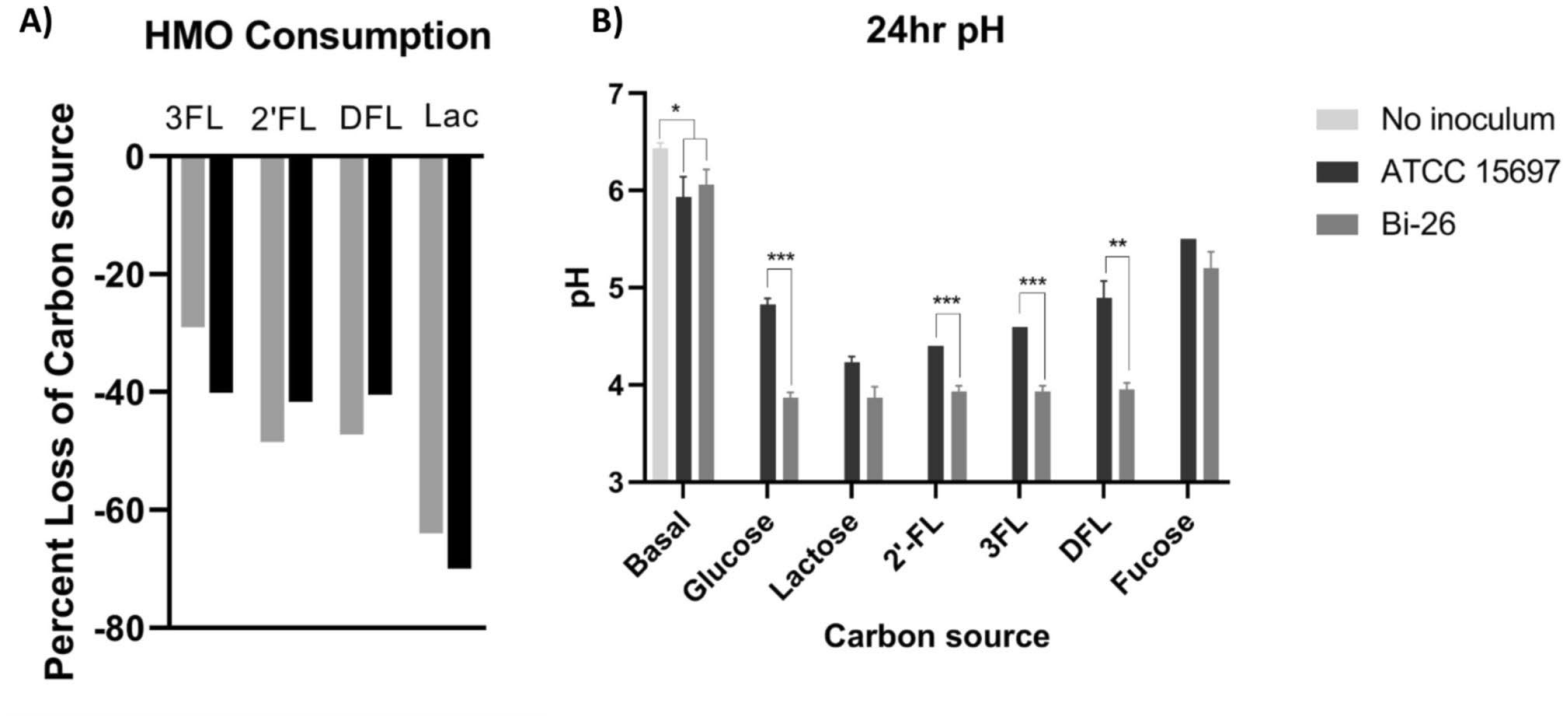
2′FL, 3FL, DFL, and lactose were measured using HPLC and consumption was calculated as a percentage of HMO or lactose lost from the media after 24 h (**A**). The pH was measured (**B**) after 24 h on each carbon source (n = 3). Significance was determined by unpaired t-test in PRISM and error bars represent 1 standard deviation from the mean. Fermentation by both strains caused acidification of the media. Overall, Bi-26 caused more acidification than the type strain except when grown on fucose (*p* value = 0.1) or lactose (*p* value = 0.0533). *p value ≤ 0.05; **p value ≤ 0.006; ***p value ≤ 0.0002.

To determine if the observed growth patterns matched the consumption of FLs, we used HPLC to measure the concentration of FLs in the media after 24 h of growth. Both strains consumed around 40–50% of the available FLs (Fig. 4A). There was no measurable difference in the amount of 2′FL, 3FL and DFL consumed by Bi-26 and the type strain after 24 h despite Bi-26 reaching a higher cell density at 24 h. When the strains were grown on fucose alone, little cell mass was created, and the final cell density was not significantly different compared to the no carbohydrate added control. pH greatly differed with Bi-26 maintaining a significantly lower terminal pH (Fig. 4B) compared to the type strain in all cases but lactose and fucose.

Overall the two strains displayed different growth rates, pH, and 24-h cell density for all the FLs tested, with Bi-26 displaying slightly better utilization of all three FL compounds.

### Analysis of metabolites produced during growth on FLs

Metabolites produced by Bi-26 and the type strain were measured when grown on FLs and lactose to determine if the observed differences in growth and media acidification were associated with secretion of different metabolites. Metabolites from the supernatant were measured at 0.10–0.20 OD_600_ (T1, entering logarithmic growth) and collected at the termination of growth at 24 h (T4). Additional timepoints for Bi-26 were taken at OD_600_ of 0.4–0.6 (T2) and OD_600_ of 1.0 (T3) to further characterize how FLs are metabolized (Supplemental Fig. 1).

**Figure 5:**
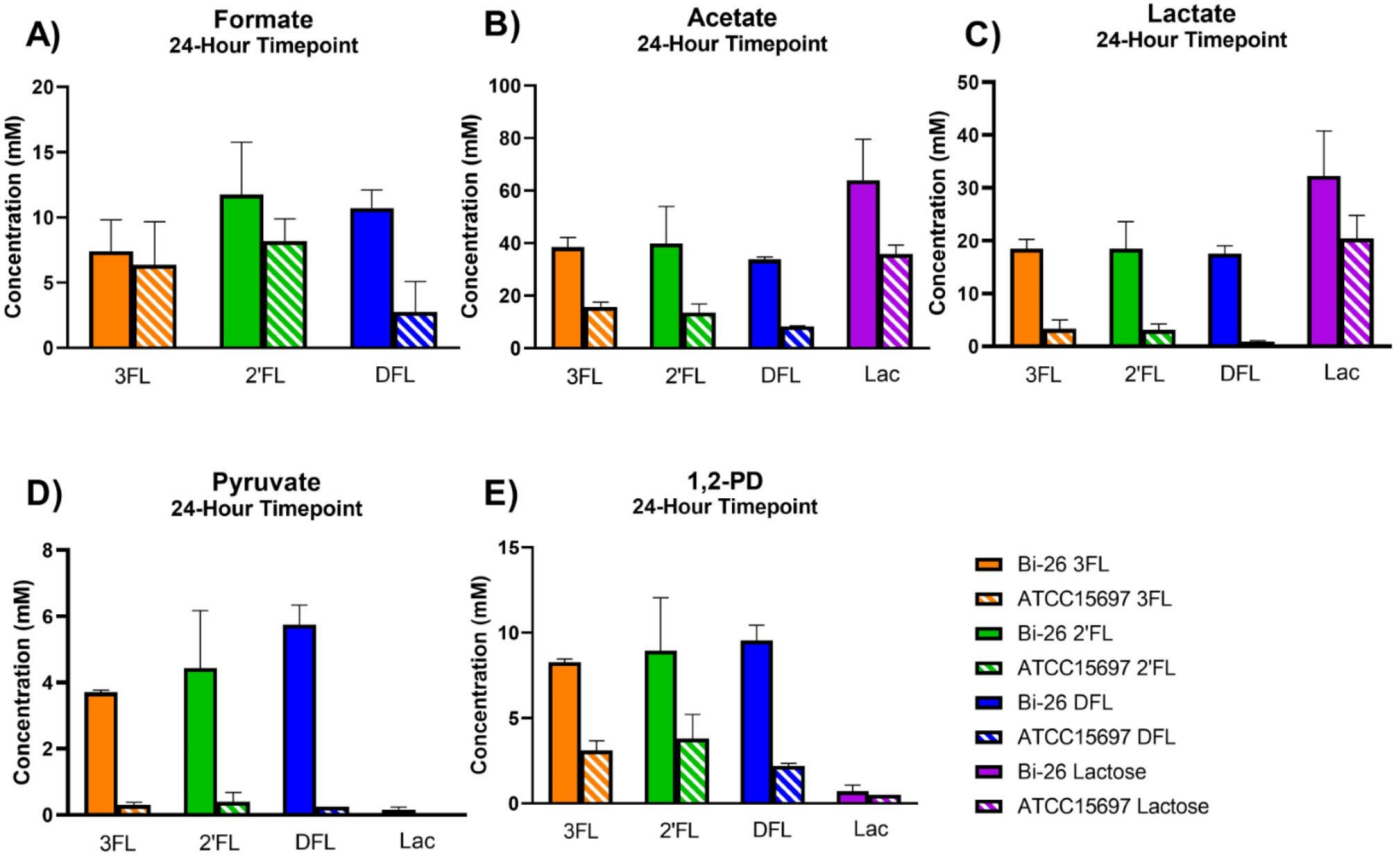
End products of fermentation grown on lactose or FLs measured via HPLC. Concentrations (mM) of formate (**A**), acetate (**B**), lactate (**C**), pyruvate (**D**), and 1,2-PD (1,2-propanediol) (**E**) at the 24-h time point are shown for ATCC 15697 and Bi-26. Two replicates of each sample were performed, and the error bars represent 1 standard deviation from the mean. Overall Bi-26 produced more lactate, acetate, formate, 1,2-PD and pyruvate when grown on FLs. Pyruvate was unique to Bi-26 and FL growth, with none being produced when grown on lactose. The data for all timepoints for Bi-26 is shown in Supplemental Fig. 1.

We first measured the secretion of fermentation end products such as formate, lactate, and acetate. Unsurprisingly, there was no significant production of fermentation end products for either strain in mBasal media with no carbon source (not shown). During growth on FLs, both strains differed significantly regarding the concentration and ratio of fermentation end products that were produced by 24 h (Fig. 5). Bi-26 produced higher concentrations of acetate, lactate and formate than the type strain. Acetate was produced at the highest concentration for both stains, but they differed in the second most abundant product being formate for the type strain and lactate for Bi-26. This resulted in a very different ratio of lactate to formate between the two strains with a ratio range of 0.33–0.52:1 for the type strain and 1.64–2.49:1 for Bi-26 (Supplemental Table 3).

Metabolism of FLs releases fucose which is further metabolized to 1,2-propandiol (1,2-PD)^19^. As expected, 1,2-PD was only detected in samples grown on FLs. In Bi-26, levels of 1,2-PD began to rise during the T2 timepoint (Supplemental Fig. 1), and the terminal concentration of 1,2-PD was twice as high in Bi-26 as compared to the type strain (Fig. 5E). Bi-26 produced the same level of 1,2-PD for all three FLs tested while the type strain produced less on DFL, likely due to poor utilization of this compound by this strain.

Pyruvate levels began to rise for Bi-26 during the T3 timepoint (Supplemental Fig. 1) for the fucosylated HMOs, while it is never present in the lactose grown samples (Fig. 5D). The type strain shows baseline pyruvate accumulation with all samples whereas Bi-26 had levels far exceeding baseline at > 3.5 mM for samples grown on FLs.

When looking at the ratio of acetate to lactate (Supplemental Table 3), Bi-26 maintained a ~ 2:1 ratio during growth on lactose, as well as on all three FL species, while for the type strain this ratio differed fivefold from 1.75:1 on lactose, to 4.37:1 on 2′FL, 4.75:1 on 3FL, and 9.33:1 ratio on DFL. This ratio inversely correlated with growth data showing the highest terminal growth (lactose) yielding the lowest ratio while the lowest terminal growth (DFL) having the highest ratio.

These results show significant differences in metabolite production during FL utilization, potentially indicating specific adaptations evident in Bi-26 to grow efficiently on FLs, the HMO species with the highest molar ratio of fucose to lactose (2:1 DFL, 1:1 other FLs).

#### Transcriptomic signatures of Bi-26 and ATCC 15697

Transcriptomics was performed to determine differences in gene expression between Bi-26 and the type strain when grown on FLs, lactose, glucose and the no carbon source control. Key metrics and statistics for all RNA-seq experiments are summarized in Supplemental Table 1.

Firstly, to determine overall differences we assessed the global patterns of transcription on Bi-26 and the type strain with principal component analysis (PCA). The PCA of Bi-26 (Supplemental Fig. 2A) showed that the different carbohydrate sources seem to be explained mostly by differences in PC1 (47% variance), and the separation of the groups on PC2 lines up with the different timepoints (21% variance), showing that most of the variation is due to carbohydrate source followed by growth stage of the culture.

The PCA analysis of the type strain showed a pattern of the FLs clustering together (Supplemental Fig. 2B), with PC1 describing carbohydrate source (61% variance), and PC2 separates no carbohydrate and lactose from the FLs (16% variance).

#### Differentially expressed genes

Differentially expressed genes (DEGs) were determined relative to growth in mBasal medium with no carbon source added and were considered significant by 1.5-fold change or higher and Benjamini–Hochberg adjusted *p* value < 0.05.

**Figure 6:**
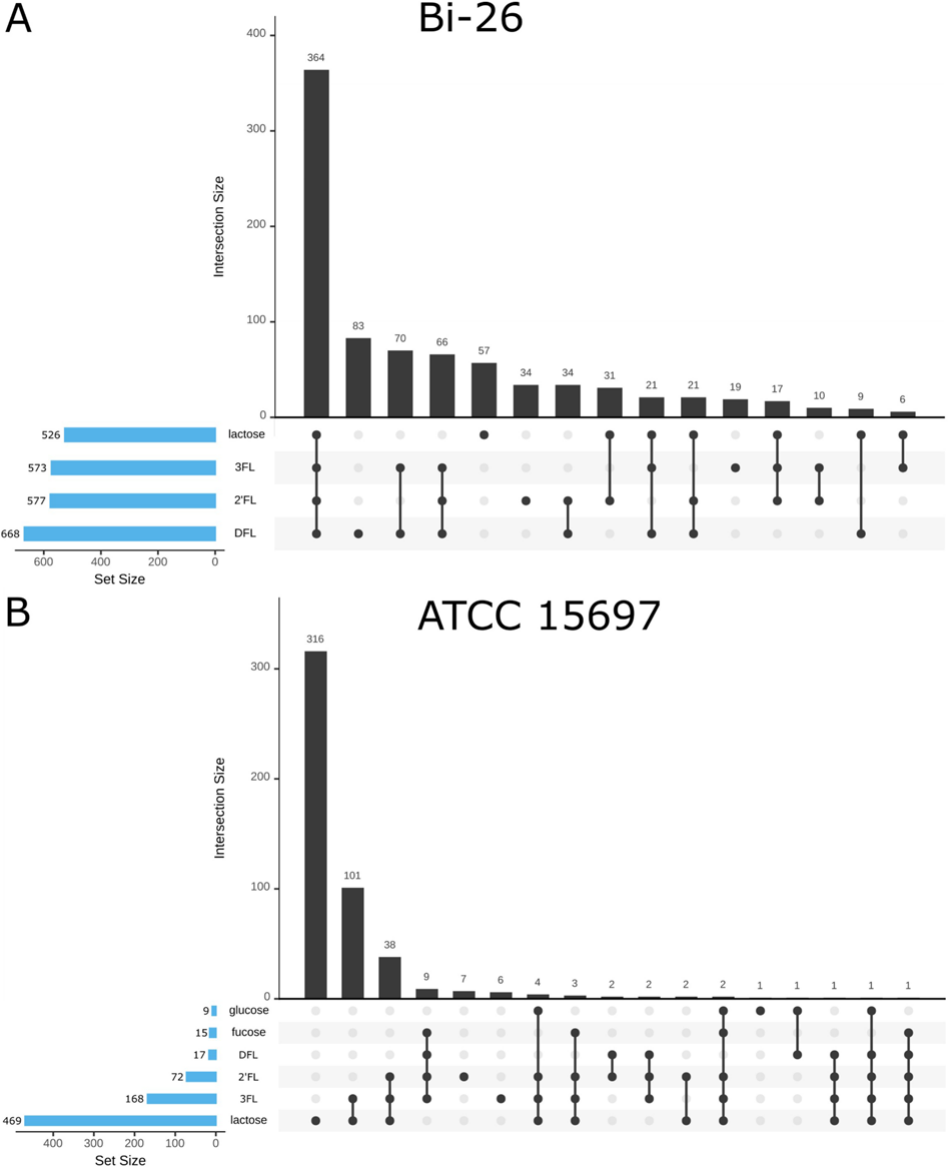
UpSet diagram of the differentially expressed genes (DEGs) for the T2 timepoint for Bi-26 (**A**) and ATCC 15697 (**B**). Total number of DEGs is shown on the left (blue bars) for each of the conditions while the number of shared genes per condition (shown by the connected dots), is shown above the black bars (significance cut-offs: Benjamini–Hochberg adjusted *p* value < 0.05; log2 > 1.5). The no carbohydrate added samples for T2 were used as the controls for the analysis.

For both strains, the total number of DEGs (up- or downregulated-) above the designated cut-off at the T2 timepoint was the largest during growth on lactose (469 for the type strain and 526 for Bi-26). This pattern of DEGs is in good agreement with the previously reported observations that lactose might play a role as one of the global regulators in *B. longum* species^26,27^ in addition to being the preferred carbon source^28^. Notably, significant differences were observed in the DEGs in the transcriptomes of 2′FL, 3FL, and DFL-grown cells between the type strain and Bi-26. The type strain showed significantly lower numbers of DEGs on FLs, as compared to lactose (72, 168, and 17 DEGs on 2′FL, 3FL, and DFL respectively), especially when grown on DFL. In contrast, the Bi-26 transcriptomes had similar numbers of DEGs on lactose and all FLs tested (Fig. 6, Supplemental Fig. 3).

This observation points to a striking difference in the global regulatory networks between these two closely related strains. It indicates that in Bi-26 the small fucosylated HMOs exert far-reaching regulatory roles arguably comparable to that of lactose. While in the type strain FLs perturb expression of a much smaller complement of genes. In particular, in the type strain DFL regulates a small set of 17 genes involved of its own uptake and utilization, while in Bi-26 a much larger number of genes (668 genes, similar to lactose) change their expression levels in the presence of this oligosaccharide (Fig. 6, Supplemental Fig. 3).

Also, of note is a very low number of DEGs evident in the type strain transcriptome on glucose, drastically lower than any other transcriptomes generated in this study (Fig. 6). This agrees with our prediction that a mobile element insertion within predicted antiterminator of BglG family (Blon_2180) had inactivated the predicted glucose PTS transporter Blon_2183. Furthermore, the small number of DEGs implies that there are no other functional transport systems for glucose uptake in the type strain making this strain technically ‘blind’ to the presence of glucose in the medium.

#### Genes involved in recognition and initial processing of 2′FL, 3FL, or DFL

**Figure 7:**
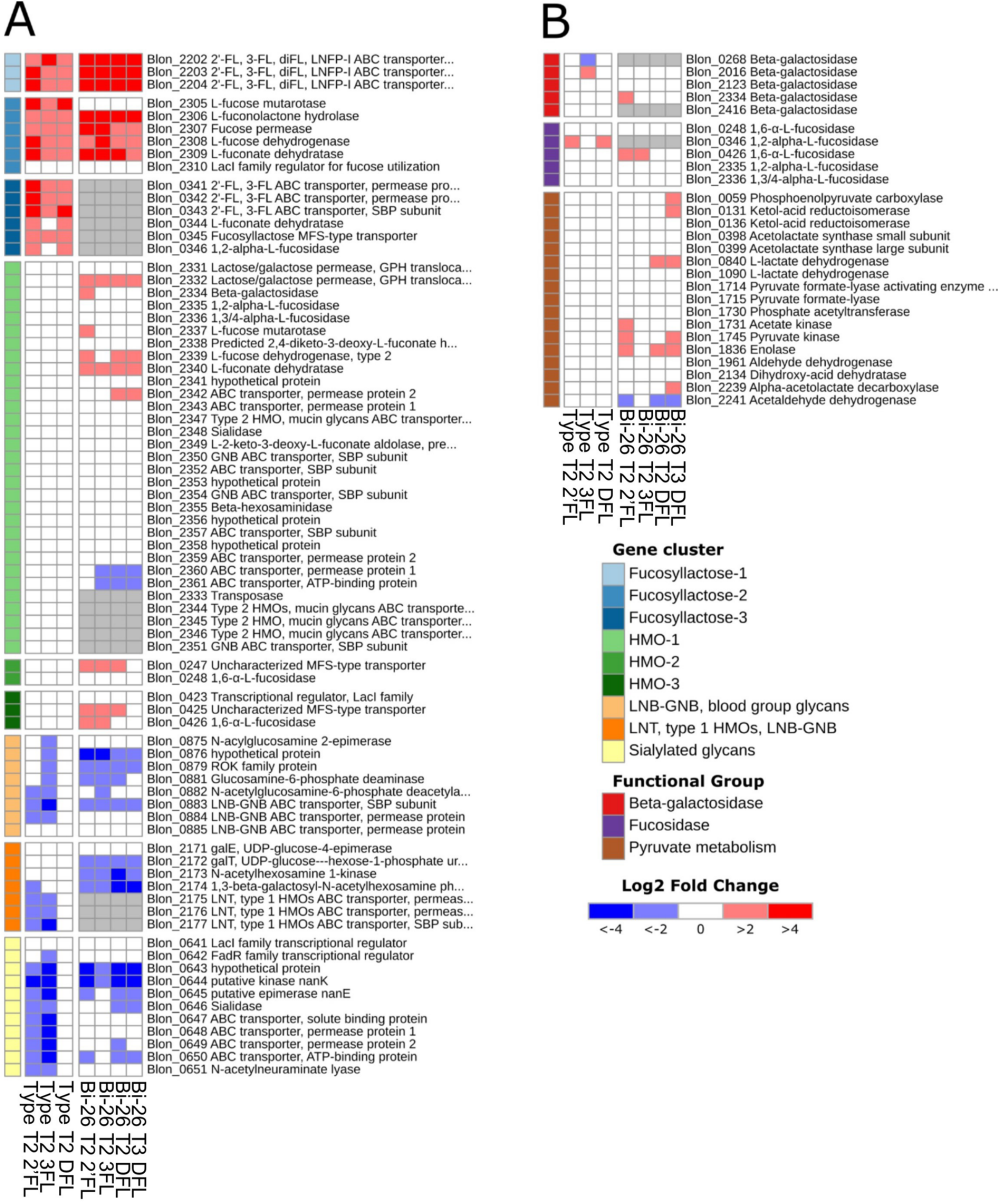
Differential expression of the HMO utilization genes within the global transcriptomes of ATCC 15697 and Bi-26 strains grown on 2′FL, 3FL, or DFL shown as log2-fold change. Transcriptomes of cultures grown in mBasal medium with no carbon source added were used as a reference. A fold change ≥ 1.5-fold and Benjamini–Hochberg adjusted *p* value < 0.05 were considered for statistical significance. Orthologous genes between the Bi-26 and ATCC 15697 are shown on the same rows and are identified by Blon IDs. (**A**) Differential expression of the gene loci implicated in recognition, internalization, and intracellular hydrolysis of fucosyllactose and several other HMO species in *B. longum* subsp. *infantis*. Genes are shown in the order on the chromosome. General HMO specificity of each locus is color-coded on the left. In gray are the orthologs of ATCC 15697 genes missing from the genome of Bi-26. Most of the missing genes are transporter systems for various HMOs, which underscores the Bi-26 apparent preference for 2′FL, 3FL, and DFL utilization. Upregulation of the L-fucose utilization genes in the HMO-1 cluster in Bi-26, but not in ATCC 15697 is another indication of a regulatory adaptation to preferential utilization of small fucosylated HMOs by Bi-26. (**B**) Differential expression of metabolic genes involved in utilization of 2′FL, 3FL, DFL and their constituent monosaccharides. See Supplemental Table 2 for explanation of abbreviations.

Since we are primarily interested in HMO utilization, we focused on genes known to be involved in HMO utilization in the type strain^19–22,29^ and other bifidobacteria^24,25,30^ to look for differences between the two strains tested. The gene list is shown in Figs. 2 and 7 and includes genes encoding the solute binding proteins (SBPs), permease and ATP-binding subunits of ABC transporters, β-galactosidases, fucosidases, enzymes of fucose utilization, ‘bifid shunt’ and Leloir pathway. In addition, we evaluated select central metabolism genes that are relevant for HMO utilization. Differential expression of important genes in Bi-26 and the type strain grown on 2′FL, 3FL, or DFL is shown in Fig. 7.

The three gene operon encoding two permease subunits and a solute binding (SBP) protein (Blon_2202–Blon_2204**)** of the main fucosyllactose ABC transporter^25,31^ was highest induced in all Bi-26 transcriptomes of 2′FL, 3FL, and DFL utilization. DFL samples showed expression patterns very similar to that in the 2′FL and 3FL transcriptomes of both the Bi-26 and the type strains (Fig. 7). However, the average increase in the transcript abundance for the genes Blon_2202, Blon_2203, Blon_2204 was approximately 25% less on DFL than on 2′FL or 3FL (74-fold average increase versus 100-fold respectively), especially pronounced for the SBP encoding gene Blon_2202.

The locus encoding fucose utilization enzymes (Blon_2305–Blon_2310)^19^ was the next most induced in the transcriptomes of both strains on all three HMO species analyzed in this study. Expression patterns were highly similar between all samples analyzed. The predicted transcriptional regulator for fucose utilization Blon_2310 peaked in the T1 sample, followed by the enzyme-encoding genes during T2 phase (Supplemental Fig. 4). In addition, all *B. infantis* genomes harbor the second fucose catabolic operon (Blon_2337–Blon_2340)^19^ located within the large HMO-1 gene cluster^20^. In Bi-26 the average transcript abundance for the genes in the first fucose utilization cluster increased over 30-fold during growth on 2′FL, 3FL or DFL, while the expression of the second cluster increased by 4.5-fold. Notably, the latter fucose utilization locus was not induced in the type strain by any of the three FL oligosaccharides tested (Fig. 7A).

Interestingly, a locus which was highly expressed in the 2′FL, 3FL, DFL transcriptomes of the type strain is missing from the Bi-26 genome altogether. This locus encodes a well-known fucosyllactose ABC transporter (Blon_0341–Blon_0343)^20,25,31^ and two enzymes of fucose metabolism: 1,2-α-fucosidase (Blon_0346) and L-fuconate dehydratase (Fig. 7A).

It is noteworthy that all top upregulated genes in the 2′FL, 3FL and DFL transcriptomes of both strains occur outside the HMO-1 cluster^21^, and that none of the genes in HMO-1 were upregulated over the cut-offs in response to any of the three FLs in the type strain. Instead, 2′FL, 3FL, and DFL induced the expression of alternative operons, distinct from the HMO-1 cluster (clusters Fucosyllactose-1, 2, and 3 in Fig. 7A). This phenomenon has been reported earlier in the type strain: loci Blon_2202–Blon_2204, Blon_2305–Blon_2309, and Blon_0341–Blon_0344 were induced by 2′FL and 3FL but—importantly—not by other HMO species^32^. These findings indicate that a dedicated pathway for fucosyllactose utilization has evolved in *B. infantis* subspecies, which does not contribute to uptake or catabolism of any other HMO species present in significant amounts in pooled breast milk. Furthermore, the results generated in this study indicate that in at least some *B. infantis* strains (exemplified by Bi-26) this pathway is used more efficiently than in others, supporting higher growth rates and potentially providing a competitive advantage over other *B. infantis* strains in infants breastfed with milk high in 2′FL and/or 3FL.

#### Expression of the glycosyl hydrolases involved in 2′FL, 3FL, DFL utilization

Once internalized, the small fucosylated HMOs are processed by glycosyl hydrolases, which are represented by multiple genes in all *B. infantis* genomes. Genomes of Bi-26 and ATCC 15697 each contain four to five genes encoding α-fucosidases and β-galactosidases. These genes showed transcriptional changes of lesser degree or none compared to the transport systems.

Two fucosidases of the five reported in the type strain were shown to have significant activity on fucosylated HMOs: Blon_2335 and Blon_2336 (Fig. 7)^33^. The three remaining α-fucosidases, Blon_0248, Blon_0426, and Blon_0346, were reported to lack activity on 2′FL or 3FL^33^. Recently Blon_0248 and Blon_0426 were characterized as 1,6-α-L-fucosidases involved in degradation of core-fucosylated N-glycans, abundant in human milk glycoproteins, such as lactoferrin, but not in HMOs^34^. Unexpectedly, in our study Blon_0346 was the only α-fucosidase upregulated during growth of the type strain on either of FLs, while neither Blon_2335, nor Blon_2336 were induced. The Bi-26 genome lacks a Blon_0346 ortholog, and in its FL transcriptomes the α-fucosidase Blon_0426 was significantly induced, while Blon_2335 and Blon_2336 were induced to a lesser extent with fold change 1.4 (slightly below cutoff) (Fig. 7B). The biological meaning of this observation is currently unclear.

In *B. infantis* two of the five β-galactosidases (Blon_2016, Blon_2334) were shown to have glycolytic activity on different HMOs^35^, including 3FL^36^. Both enzymes were shown to be transcribed constitutively^35^. In our study Blon_2016 showed upregulation above the basal expression level on 3FL, but not on 2′FL in the type strain, while Blon_2334 showed upregulation on 2′FL but not 3FL in Bi-26 (Fig. 7B). Of the five β-galactosidases genes described in type strain, Bi-26 possesses the orthologs for only three. The lower gene copy number of both the α-fucosidases and β-galactosidases evident in Bi-26 is in good agreement with a slight tendency toward genome minimization we observe in Bi-26 and the closely related *B. infantis* strains (Fig. 1).

#### Expression of central carbohydrate metabolic pathways during growth on fucosyllactose

Unlike the uptake transport systems for fucosyllactose, the genes encoding enzymes of catabolic pathways converting FL-derived monosaccharides to the intermediates of central carbohydrate metabolism showed transcriptional changes of lesser degree or none. We attempted to discern if any metabolic genes or pathways were differentially expressed between 2′FL, 3FL and DFL by comparing directly the Bi-26 transcriptomes obtained on each of these HMOs at different growth stages. There is a complete absence of the differentially expressed (DE) genes between the conditions 2′FL, 3FL and DFL with Bi-26. Such similar global expression patterns observed on all three FLs indicate that metabolic routes of their uptake and utilization in Bi-26 are nearly identical, with a few important exceptions discussed below.

Three enzymes of pyruvate metabolism [ketol-acid reductoisomerase (EC 1.1.1.86, ilvC), alpha-acetolactate decarboxylase (EC 4.1.1.5, ALDC), and to a lesser extent, acetolactate synthase (EC 2.2.1.6, ALS)] were slightly but significantly upregulated in the DFL transcriptomes, especially at the T3 timepoint, but not in either 2′FL, or 3FL transcriptomes (Fig. 7B). Acetolactate synthase catalyzes pyruvate conversion to (S)-2-acetolactate, a precursor in the biosynthesis of L-valine and L-leucine. This enzyme can also transfer the acetaldehyde from pyruvate to 2-oxobutanoate, forming 2-aceto-2-hydroxybutanoate, a reaction in the biosynthesis of L-isoleucine^37,38^. Both compounds, (S)-2-acetolactate and 2-aceto-2-hydroxybutanoate, are next utilized by ketol-acid reductoisomerase (NADP+), the enzyme with the highest DE level among the three (1.8-fold, *p* < 0.05) in the pathways leading to branch-chain amino acids L-valine, L-leucine, and L-isoleucine^39,40^. Alternatively, (S)-2-acetolactate can be dissipated via conversion to (R)-acetoin by the action of alpha-acetolactate decarboxylase, another enzyme of the three upregulated during Bi-26 growth on DFL (Figs. 2, 7B). Notably, all three enzymes dissipate pyruvate without acidifying growth medium or excreting any end products other than volatile CO_2_ and acetoin (Fig. 2). Activation of these pathways sheds light onto the pyruvate excretion observed in Bi-26 in this study (above), as both phenomena lead to pyruvate dissipation during growth on fucosyllactose. Considering the late onset of both processes, they appear to be a specific adaptation in Bi-26 for maintaining ATP generation when the classic fermentation pathways slow down, as the excretion of their end products (acetate, lactate, formate, ethanol) is arrested by significant accumulation of these compounds in the spent medium.

We hypothesize that under these conditions, any dissipation of pyruvate (via its efflux driven by product gradient or/and its enzymatic conversions) can drive forward the pyruvate kinase (EC 2.7.1.40) reaction facilitating ATP synthesis from phosphoenolpyruvate (Fig. 2). During growth on fucosyllactose this adaptation is important, as the fucosyl moiety of FLs is catabolized to pyruvate (and 1,2-PD) as the end products via anaerobic non-phosphorylating pathway characteristic of bifidobacteria^19,41^. This ‘excessive’ pyruvate generation could potentially inhibit the essential ATP-generating pyruvate kinase reaction above (Fig. 2). The differences in the pyruvate metabolism detected in this study between the Bi-26 and the type strain likely explain the increased growth of Bi-26 on FLs and underscore specific adaptation of this *B. infantis* strain to the utilization of small fucosylated HMOs.

#### Gene loci significantly down-regulated during growth on 2′FL, 3FL, or DFL

Expression of several HMO related clusters was down-regulated in the transcriptomes of FLs as compared to the transcriptome obtained during growth on mBasal media with no carbon added. The top HMO-related loci with the highest degree of repression by FLs are described below. These clusters are displayed in Fig. 7A.

Utilization of type 1 HMOs represented by Blon_2171–Blon_2177, encodes the enzymes of the modified Leloir-like pathway for processing lacto-N-biose (LNB) and galacto-N-biose (GNB)^42^ and an ABC transporter solute binding subunit Blon_2177 with high affinity for type 1 HMOs: Lacto-N-Tetraose (LNT), lacto-N-hexaose (LNH), lacto-N-octaose (LNO)^43,44^ and related glycans, including sialylated LNT and Lacto-N-Neotetraose (LNnT), GNB and LNB^30^. In our study in the type strain the entire cluster was significantly downregulated during growth on 2′FL, 3FL, but not during growth on DFL. In Bi-26 the transporter Blon_2175-2177 is missing, while the remainder of the locus is downregulated to the same extent by all three species of fucosyllactose.

LNB-GNB cluster (Blon_0875–Blon_0885) encodes an ABC transporter with solute binding subunit Blon_0883 specific for LNB as well as GNB and three enzymes of hexosamine metabolism. The cluster was shown to be induced by HMO, LNT and LNnT^44,45^, however, to be imported via Blon_0883, these inducing type 1 HMOs need to be hydrolyzed first to disaccharide LNB. Since extracellular glycosylases are not characteristic of *B. infantis*, this locus could be involved in scavenging LNB generated from extracellular HMO degradation by other bifidobacterial species^46,47^ or other intestinal bacteria^44^. In our study, the LNB-GNB cluster in the type strain was strongly downregulated during growth on 3FL, while a smaller number of genes was inhibited by 2′FL, and DFL did not inhibit this cluster. In contrast the LNB-GNB cluster was downregulated in Bi-26 to the same extent by all three FLs (Fig. 7A).

The sialic acid catabolism cluster includes genes encoding sialidase NanH1 (Blon_0646), *N*-acetyl neuraminic acid lyase (Blon_0651), putative kinase NanK, epimerase NanE, and predicted sialic acid ABC transporter (Blon_0647–Blon_0650). Albeit originally believed to be involved in HMO utilization^20^, the role of this cluster in *B. infantis* is now considered to be in utilization of sialylated glycans other than HMOs, e.g. milk glycopeptides or sialylated mucins^48^. Pronounced downregulation of (or parts of) this cluster by 2′FL and 3FL (and by DFL in Bi-26 strain) evident in our study might indicate that fucosyllactose is prioritized by *B. infantis* over these potential substrates as well.

In Bi-26 all three FL compounds demonstrated the same pattern of downregulation of competing HMO utilization genes. In the type strain only 3FL demonstrated the same level of regulatory capacity, while 2′FL inhibited a smaller number of genes (Fig. 7A). Furthermore, DFL did not inhibit any genes in the type strain (Fig. 7A), except for two hypothetical proteins. These differences are another indication of a regulatory adaptation for preferential utilization of small fucosylated HMOs by Bi-26.

### Complement of HMO transporters in Bi-26 and the type strain

ABC transporters generally contain an extracellular component (SBP, solute binding protein), which binds the target substrates and facilitates their transfer to membrane-embedded permease subunits of the transporter for internalization^49^. SBPs are the major determinants of transporter specificity^49^ and the complement of SPB encoding genes in a genome predicts the spectrum of substrates available to the organism. The type strain genome encodes 20 genes of family 1 SBPs (pfam0147), which is among the highest SBP copy numbers among all sequenced bifidobacterial genomes^44^. Half of the SBPs were determined to bind mammalian glycans, including various types of HMOs^44^. Analysis of the presence or absence of orthologous of these genes showed that four SBPs are missing from the Bi-26 genome: Blon_2344, Blon_2351, Blon_0343 and Blon_2177 (Fig. 7A). Notably, three of them have one or more related SPBs with similar substrate specificity remaining in the Bi-26 genome (Fig. 7A). In particular, Blon_2344 and Blon_2347 specifically recognize type 2 glycans binding linear and branched polylactosamines, as well as lacto-n-neotetraose (LNnT) and difucosyl lacto-N-hexaose^44^. While Blon_2344 is absent in Bi-26, Blon_2347 remains. Likewise, Blon_2351, Blon_2350 and Blon_2354 specifically detect GNB, supporting their role in binding and/or import of mucin oligosaccharides^44^. While Blon_2351 is no longer present in Bi-26, SBP subunits Blon_2350 and Blon_2354 remain. Likewise, out of two fucosyllactose-specific ABC transporters characterized in the type strain, only one is present in Bi-26 genome: Blon_2202–2204, while Blon_0341–0343 is not (Fig. 7A). Importantly, Blon_2202–2204 has a wider substrate specificity than Blon_0341–0343. While both transports efficiently internalize 2′FL and 3FL, Blon_2202–2204 supports utilization of additional fucosylated HMOs, including DFL and lacto-N-fucopentaose I^31^. This broader specificity may explain why Blon_2202 homologs are found in two times the number of publicly available bifidobacterial genomes than Blon_0343. Furthermore, Blon_2202 was found to be significantly more prevalent in fecal metagenomic samples of infants living in Japan, Malawi, and Venezuela^31,50^.

Such pattern of SPB gene loss potentially reflects a tendency towards genome size reduction in Bi-26 without significantly limiting the range of HMO substrates available for uptake. The only exception is the Blon_2175–Blon_2177 transporter with high affinity for LNT and longer type 1 HMOs^43,44^. Its absence from the Bi-26 genome (Fig. 7A) likely renders this strain incapable of internalizing these HMOs whole. However, the presence of an intact LNB-GNB transporter Blon_0883–Blon_0885 in Bi-26 (Fig. 7A) indicates that these type 1 HMOs could in fact be consumed by Bi-26 in the presence of other intestinal bacteria capable of extracellular HMO degradation to LNB or other constituents^44,46,47^.

## Discussion

The ability to utilize HMOs differs between the strains of infant-associated bifidobacterial species. While in *B. breve*,* B. longum* subsp. *longum*, and *B. kashiwanohense* the capacity for uptake and catabolism of HMOs is limited to specific strains and to selected types of HMOs^18,30,51^, in *B. infantis* strong growth on a wide range of HMOs is considered characteristic of the entire subspecies^20,52,53^. The detailed analysis of molecular mechanisms of HMO utilization has been addressed largely in a single representative of *B. infantis* subspecies, the type strain ATCC 15697, with a few exceptions^20,24^ leading to the underestimation of the genetic and metabolic diversity present within the *B. infantis* subspecies as a whole. In this study we show that at least two distinct strategies for FL utilization are evident within the *B. infantis* subspecies. While the type strain is a generalist, that was shown to consume different types of HMOs simultaneously and equally well without a preference for any neutral or fucosylated HMO^8,22,33,52^, we propose that Bi-26 is adapted to quickly internalize small fucosylated HMOs. This could provide a competitive advantage via rapidly depleting the preferred substrates (2′FL, 3FL, DFL) and preventing other bifidobacteria from accessing them. Additionally, through metabolism of small, fucosylated HMOs, Bi-26 creates a low pH environment that may exclude potential pathogens. Our findings support this conclusion: Bi-26 reduced genome, differences between Bi-26 and the type strain in growth rates and final cell density at 24 h during growth on FLs, differences in excreted fermentation products, and global transcriptional responses of the two strains to each of FLs species.

Small fucosylated HMOs, the focus of this study, present a bit of a paradox. They are readily available in human breast milk (aside from milk derived from non-secretor or/and Lewis negative mothers^54^) with 2′FL being the most abundant HMO type^8,9,11^ representing up to 45% of the total HMO content in individual breast milk^12^. Yet, fucosyllactose-utilizing strains are rare among breast-fed infant-isolated bifidobacteria outside *B. infantis*,* B. kashiwanohense*, and *B. bifidum*^19,30,51^. Indeed out of 17 *B. longum* subsp. *longum* strains previously tested^30^, only one could grow on 2′FL or 3FL, while all grew well on LNT^30^. Likewise, 2′FL supported vigorous growth of merely 2 *B breve* strains out of 24 tested^51^ while all grew on LNT. We hypothesize that 2′FL, 3FL, and DFL present a challenge to use as a carbon source, as this group has the highest fucose to lactose molar ratio among all HMO species: 2:1 in tetrasaccharide DFL, and 1:1 in trisaccharides 2′FL and 3FL. Fucose is a poor growth substrate for all bifidobacteria, as this taxonomic group lacks the classic aerobic route of fucose catabolism via phosphorylated intermediates^55,56^ and instead possess the anaerobic non-phosphorylated pathway that yields no ATP^19,41,57^. None of bifidobacterial strains tested demonstrated significant growth on L-fucose^18^. In *B. longum* subsp. *infantis* some growth with L-fucose as sole carbohydrate source could be enabled only in the presence of trace amounts of glucose (0.4 mM)^19,58^. In *B. breve* UCC2003 the ‘substrate utilization without growth’ phenotype was observed with L-fucose as sole carbohydrate source. A marked decrease in the concentration of L-fucose with concurrent increase in its degradation products (acetate, formate and 1,2-PD) was not accompanied by any change in OD^18^. Likewise, in our study no significant growth on L-fucose as the sole carbon source was observed for both strains (Fig. 3B).

The non-phosphorylated fucose catabolic route is not yet fully elucidated in bifidobacteria^19,23^, which in turn complicates the analysis of FLs utilization in these organisms. This is especially true of *B. infantis*, which relies on HMO consumption strategy based on the import of intact HMO structures and their intracellular enzymatic degradation^22,32,59^. Figure 2 illustrates the current limited understanding of the concurrent assimilation of the FLs constituents L-fucose and lactose. Notably, pyruvate is the central cross-point intermediate in utilization of both, carrying the major carbon flux in FL utilization. This situation is unique for bifidobacteria and is determined by the fact that the so-called fructose 6-phosphate or bifid shunt is the only pathway involved in energy recruitment from lactose within the limited central metabolic network of these non-respiring obligate anaerobes^29^. The central position of pyruvate in fucosyllactose utilization by *B. infantis* prompted us to analyze this compound along with the ‘classic’ end fermentation products: acetate, lactate, and formate. The striking difference observed between the Bi-26 and type strains grown on each FL was the significant pyruvate accumulation in the spent medium in the former but not in the latter strain. Importantly, no pyruvate was detected in cultures grown on lactose as the sole carbon source for either strain. Quantitatively pyruvate accumulation was highest during Bi-26 growth on DFL (~ 5.8 mM at 24 h) and less on 2′FL and 3FL: 3.7 and 4.4 mM respectively, which agrees with different fucose fractions in these fucosyllactose species. The excreted pyruvate levels were comparable to the 1,2-PD molar levels accumulated in the same samples (from 8 to 9.8 mM for all FLs). These numbers are quite high because 30 mM DFL, or 40 mM 2′FL or 3FL (20 g/L of each oligosaccharide) were added to each culture at the start of fermentations, with about half of them utilized in 24 h. 1,2-PD is the known product of L-fucose catabolism and can be used to quantify the fraction of fucose catabolized in each culture. Since about half of available DFL (15 mM) was consumed by Bi-26 in 24 h (Fig. 4A), twice the molar amount of the fucosyl moiety in DFL (30 mM) must have been processed by the cells. As shown in Fig. 5E, about 10 mM of that was excreted as 1,2-PD, indicating that about a third of available fucose was catabolized by Bi-26 to 1,2-PD and pyruvate. The levels of excreted pyruvate at ~ 5.8 mM (Fig. 5D) indicate that about half of the pyruvate generated in this process was excreted and not incorporated into the biomass. Significantly lower levels of 1,2-PD excreted by the type strain (as compared to Bi-26) during growth on any of the three FL compounds indicate that a smaller fraction of FL fucosyl moiety is likely catabolized by this strain to pyruvate and 1,2-PD. The fate of the remainder of the fucosyl moiety in the type strain or in Bi-26 was not pursued in this study. However, from out earlier work^24^ we know that at least a part of it is excreted by Bi-26 as L-fucose during growth on 2′FL^24^ and that the amounts of 1,2-PD and L-fucose accumulating in the spent medium inversely correlated under these conditions (not shown). In the absence of any fucose catabolic pathways in *Bifidobacterium* genera other than the non-phosphorylated anaerobic route, the L-fucose monosaccharide excretion and its catabolism to pyruvate and 1,2-PD are the only two alternative fates of the fucosyl moiety in FLs (or other fucosylated HMOs) conceivable to date (illustrated schematically in Fig. 2). Currently the prevailing opinion in the literature is that *B. infantis* does not release L-fucose or other monosaccharides outside the cell, allegedly preventing cross-feeding to other gut species^32,59^. However, L-fucose accumulation in the spent medium during the *B. infantis* growth on fucosylated HMOs has been reported in the literature, even if in passing^24,33,60^. Although beyond the scope of this study, this phenomenon warrants further investigation, as fucose metabolism is highly relevant in vivo in the mammalian host^61,62^, an environment where fucose is an abundant component of mucin and milk glycans and glycoproteins, potentially available to gut microbiota^63,64^ and is one of important mediators of host-microbe symbiosis^61,64–68^.

Pyruvate excretion by *B. infants* during growth on HMOs has not been reported in the literature, except as a brief mention in an earlier publication from our group^24^. In addition to pyruvate excretion by Bi-26 (Fig. 5D, Supplemental Fig. 1E), we also observed an activation of several pathways of pyruvate dissipation in the DFL transcriptome of this strain, especially at the T3 timepoint (Fig. 7B). This pattern of a slight but significant upregulation of several pyruvate metabolism genes in Bi-26 indicates that pyruvate can be converted to a volatile compound R-acetoin and/or to S-2-acetolactate and 2-aceto-2-hydroxybutanoate, two precursors in the pathways leading to branch-chain amino acids L-valine, L-leucine, and L-isoleucine^37,38^, which have the potential to serve as intracellular pyruvate sinks (Fig. 2).

Both processes, the internal enzymatic dissipation and the excretion of pyruvate, have the potential to be an adaptation to growing in the infant gut where acetate has been shown to accumulate to high concentrations. With acetate at high levels shown in exclusively breast-fed babies (11:1 acetate:lactate)^69^ the excretion of acetate against its steep gradient becomes inefficient, impeding the carbon flux through the bifid shunt and ATP generation. The tolerance of bifidobacteria towards the end products (lactate and acetate) is known to be low^70,71^, as compared to lactococci or lactobacilli, for example. Cessation of growth for *B. breve*,* B. longum*, and *B. infantis* was reported to occur in the medium containing 12–18 g/L of both lactate and acetate^36^ under pH-controlled conditions, while growth rate decline was observed at even lower concentrations of these end products, at 10 g/L^71^. For comparison, for *Lactobacillus sp*. critical lactate concentrations lie in the range of 60 to 90 g/L^36^. Switching to pyruvate excretion as an additional fermentation end product under these conditions could be a valuable strategy of energy generation.

End-metabolites of bifidobacteria and other lactic acid bacteria (LABs) in human gut have been shown to serve as substrates for secondary degraders such as butyrate-producing colonic bacteria^72–76^. We hypothesize that the pyruvate and L-fucose excretion by *B. infantis* Bi-26 reported in this study may represent additional potentially important factors in the ecological role of bifidobacteria in infant gut acting directly on the human colon epithelial cells or through cross-feeding to yield the health benefits associated with infant gut microbiota if dominated by *B. infantis.*

The theoretical molar ratio of acetate to lactate of about 1.5:1 is generally observed in bifidobacteria when hexose is supplied as a fermentable substrate in batch culture, but this ratio is not always obtained with other growth substrates or culture conditions^77^. For example changes in end product formation in species of *Bifidobacterium* have been corelated with the specific rate of sugar consumption^78^. When the specific sugar consumption rate increased, relatively more lactic acid and less acetic acid, formic acid, and ethanol were produced, and vice versa^78^. In our experiments, however, the rate of growth and the total amounts of oligosaccharides consumed in 24 h differed modestly between ATCC 15697 and Bi-26 (Figs. 3, 4) and cannot fully explain the much larger differences in acetate:lactate ratios between the strains (Supplemental Table 3).

Higher ratio of acetate:lactate ratio has been reported to occur due to phosphoroclastic splitting of pyruvate (derived from carbons 4, 5, and 6 of hexose) to acetyl phosphate and formate^79,80^. In this regard, the sharp increase in acetate and formate levels over lactate in ATCC 15697 (especially during growth on DFL) is likely due to increased conversion of the fucose-derived pyruvate into acetyl phosphate and formate. In contrast, Bi-26 employs a different strategy to dispose of the fucose-derived pyruvate: namely, via its excretion and dissipation to (R )-acetoin and/or intermediates of branched-chain amino acid biosynthesis (Fig. 2). The large differences in end product production are reflected also in the significant terminal pH difference (pH = 5.13 in ATCC 15697 vs. pH = 3.89 in Bi-26 at 24 h on DFL), because acetate, which is produced predominantly by ATCC 15697 (Supplemental Table 3), has the lowest acid strength of the four organics acids: acetic, pKa = 4.756; lactic, pKa = 3.86; formic, pKa = 3.745; pyruvic, pKa = 2.50)^81^.

2′-FL and 3FL are important for the colonization of bifidobacteria in the infant gut^3,54^. Supplementation of 2′FL by non-secretor mothers may be able to provide those same benefits. Additionally, with the rise in caesarian births and decreasing diversity in the microbiome^82^, supplementation with *B. infantis* strains utilizing different metabolic strategies of fucosyllactose utilization may be beneficial to infants that would not be exposed to it naturally.

Isolates capable of efficient utilization of fucosyllactose have been reported in other species of Bifidobacteria, including *B. breve*^51^, *B. longum*^30^, *B. pseudocatenulatum*^25^, and *B. kashiwanohense*^18,19^; and the genes controlling fucosyllactose uptake, intracellular hydrolysis, and L-fucose utilization have been identified. It would be also important to test whether these FL utilizers in other Bifidobacterial species possess the metabolic and regulatory networks modifications similar to those reported in this study for *B. infantis* Bi-26, which facilitate efficient growth on this often abundant but challenging substrate in mother’s milk.

In conclusion the results of this study show the importance of strain specific properties even within a subspecies in a narrow niche environment. Even though HMO utilization is a characteristic of *B. infantis* subspecies, we see two very different strategies for utilization by Bi-26 and the type strain. Bi-26 has evolved an ability to quickly metabolize FLs while preserving homeostasis within the cell, whereas the type strain has maintained all genes needed for metabolism of all HMOs. Current methods of microbiome research lack the resolution to discern strain specific differences that shape the complex network of host-microbial interactions in the human gut. Even within the simplest example, the infant gut, where microbiome complexity is a fraction of that in an adult, the impact of strain-specific metabolic variations on this complex ecosystem is not well understood. Defining individual strain’s roles within the complex system will be essential for understanding the numerous interactions affecting host health throughout life and can be used as a guide for health promoting supplementation.

## Materials and methods

### Genomic comparisons

We examined ~ 330 public *B. longum* genomes available in NCBI^83^ and PATRIC^84^ databases (as of October 2019) to determine species and subspecies classifications using PATRIC’s pipeline for phylogenetic tree construction^84^. Briefly, 100 core proteins (encoded by single copy genes present in all included genomes) were aligned using RAxML^85^. From this tree, we identified 36 genomes that clustered with the type strain *B. longum* subsp. *infantis* ATCC 15697 and 8 genomes that formed a distinct clade with *B. longum* subsp. *suis* type strain LMG 21814, assuming all other genomes to fall into *B. longum* subsp. *longum* subspecies. We then created a smaller representative core genome tree for the *B. longum* species (Fig. 1) with 30 of the identified *B. infantis* genomes with acceptable quality, all publicly available genomes that clustered with *B. longum* subsp. *suis* type strain, and 30 diverse represented genomes of *B longum* subsp. *longum*, chosen for the maximum representation of the inter-subspecies diversity from ~ 300 nonredundant genomes of the *B. longum* subsp. *longum* subspecies available in PATRIC database. This final tree was built with RAxML^85^ in the Phylogenetic Tree Building Service of the PATRIC database^84^, described in detail here^86^. It was based on the alignment of the concatenated amino acid and nucleotide sequences of 500 core proteins families. Support values were generated using 100 rounds of the ‘Rapid’ bootstrapping option^87^ of RaxML.

### Bacterial strains and culture conditions

We used *B. longum* subsp. *infantis* strain Bi-26 (Bi-26) obtained from the DuPont Global Culture Collection (Germany) and *B. longum* subsp. *infantis* strain ATCC 15697 (type strain) obtained from the American Type Culture Collection (Manassas, VA). Both strains were maintained in Man Rogosa and Sharpe Medium (MRS) supplemented with L-cysteine HCl (0.05% w/v) at 37 °C under anaerobic conditions achieved using the BD GasPak EZ system. For all experiments, we modified the basal media from Asakuma et al.^8^ to create modified basal media (mBasal) containing: 10 g/L BD peptone, 2 g/L yeast extract, 5 g/L NaCl, 2 g/L diammonium citrate, 0.2 g/L magnesium sulfate, 2 g/L dipotassium hydrogen phosphate. The pH was adjusted to 6.4. After autoclaving, we added filter-sterilized (0.2uM, PES membrane) 0.05% (w/v) L-cysteine HCl and 2% (w/v) of the indicated carbon source.

Prior to all experiments, stock cultures were grown overnight on MRS before being transferred to mBasal media. The cells were washed by centrifugation at 5000 rcf for 5 min, the supernatant was removed, and the cells were then resuspended in equal volume of mBasal media. All experiments were set-up under anaerobic conditions inside a temperature and humidity-controlled glove box (Plas-Labs, Lansing, MI) fitted with a palladium catalyst and mixed gas (5% CO_2_, 10% H_2_, 85% N_2_). Prior to inoculation, the media was acclimated overnight in the anaerobic chamber.

### Bacterial growth in vitro on HMO

Both strains were tested for growth on glucose, lactose, 2′-fucosyllactose (2′FL), 3-fucosyllactose (3FL), di-fucosyllactose (DFL), or fucose as the sole source of carbon. CARE4U 2′FL, 3FL, and DFL (FLs) were obtained from DuPont N&B, Ghent, Belgium. Glucose and lactose were obtained from Sigma-Aldrich (St. Louis, MO). All growth experiments were conducted in triplicate in Hungate tubes with 5 mL of mBasal media supplemented with 2% (w/v) of each carbon source and an initial inoculum of 0.05 optical density at 600 nm (OD_600_). All tubes were incubated at 37 °C for 24 h. Optical density readings were taken every 1–2 h for 24 h. The initial and final pH was measured using Thermo Scientifics Orion star A211 pH meter. The relationships among 24 h OD_600_ readings were evaluated by two-tailed, student’s t-test in PRISM version 8 (GraphPad, San Diego, CA, US).

### Collection of samples for metabolite analysis and RNA sequencing

On carbon substrates supporting robust growth (lactose, 2′FL, 3FL, DFL for Bi-26) the samples for RNA sequencing and metabolite analysis were collected at the following time points: T1 at 0.10–0.20 OD_600_ entering logarithmic growth; T2 at 0.30–0.55 OD_600_ early to middle logarithmic growth phase, and T3 at 0.73–1.05 OD_600_ approaching late logarithmic phase, prior to entering stationary phase. In addition, the T4 samples were collected at the termination of growth at 24 h for metabolite analysis (not used for RNAseq). On poor carbon sources that did not support the classic growth curve (no carbon, fucose) with very little OD_600_ changes the sampling was done twice: upon reaching 0.1–0.2 OD_600_ (T1) and at 12 h after inoculation (T2/T3). All three time points were processed for RNAseq analysis for Bi-26, the focus of this study, while a single time point for each carbon source was analyzed for ATCC15697, corresponding to T2. Accurate sampling is essential for comparison of cell growth and physiology between two different strains grown on 6 different carbon sources. Significantly different growth profiles demonstrated by Bi-26 and ATCC15697 on some substrates (e.g. 2-FL, DFL, glucose) made the use of matching OD_600_ or growth times insufficient for proper sampling. In such cases matching of samples for comparisons was aided by the use of global expression profiles.

Duplicate, independent experiments were conducted for all conditions. The cells were centrifuged at 5000 rcf for 5 min. The supernatant was removed and filter sterilized (0.2 µM, PES membrane) before being frozen at − 20 °C for later metabolite analysis. The pelleted cells were resuspended in RNAprotect Bacterial reagent (Qiagen) following the manufacturer’s protocols. The resulting cell pellets were frozen at − 80 °C. Cell pellets from Bi-26 T1, T2, T3, and type strain T2 were sent to Genewiz (South Plainfield, NJ) for RNA isolation and sequencing.

### Metabolite and carbon source analysis using ion chromatography and HPLC

Samples were tested for production of lactic acid, formic acid, acetate, lactate and pyruvate for T1 and 24-h (T4) timepoints for both strains. Samples of Bi-26 T2 and T3 were also tested. Additionally, those samples were tested for the carbon sources lactose, 2′FL, 3FL and DFL. Bi-26 HPLC was able to quantify all these compounds except for formic acid which was measured using ion chromatography.

The metabolite samples were diluted to fall into the calibration range with HPLC-grade water obtained from in-house water purification system (Barnstead GenPure Pro, Thermo Scientific, Waltham, MA, USA). Optima LC–MS Grade formic acid (Fisher Scientific) standards were diluted with HPLC-grade water for preparation of the calibration curve. The concentration of formic acid in the samples was measured using a Thermo Dionex ICS5000 ion chromatography system (Thermo Scientific) fitted with A Dionex IonPac™ AS19-4 µm column (2 × 250 mm, Thermo Scientific) with a guard column, Donex IonPac™ AG19–4 µm, 2 × 50 mm. The flow rate was 0.25 mL/min and an eluent generator with potassium hydroxide solution was used to generate the mobile phase. The gradient (in mM KOH) is as follows: 0–8 min: 5 mM; 8–8.5 min: 100 mM; 8.5–14 min: 100 mM; 14–14.5 min: 5 mM; 14.5–20 min: 5 mM. The suppressor (AERS-2 mm) was set to 38 mA. The column temperature was maintained at 30 °C and the sample injection volume was 12 µL. An external calibration curve was used to calculate the concentration of formic acid in the samples using linear regression.

HPLC with evaporative light scattering detection was used to identify the carbohydrates. Metabolite samples were further diluted with acetonitrile (1:1 acetonitrile:sample) to match the mobile phase conditions. Fucose and lactose standards (≥ 99% purity) were obtained from Sigma-Aldrich. The CARE4U 2′FL, 3FL along with DFL standards were received from DuPont N&B. A mixed standard of fucose, lactose, 2′FL and DFL was prepared in acetonitrile:water (1:1). A separate standard of 3FL was prepared due to poor resolution from the 2′FL standards. This did not affect sample analysis since the two analytes were not present in the same sample. Analysis was performed using a Thermo Vanquish Flex UHPLC system (Thermo Scientific) with a Shimadzu ELSD-LTII Evaporative Light Scattering Detector (ELSD, Shimadzu Scientific Instruments, Columbia, MD, USA). An XBridge Amide column (2.1 × 50 mm, Waters Corp, Milford, MA, USA) was connected in series to a Luna Omega Sugar column (2.1 × 150 mm, Phenomenex, Torrance, CA, USA). The column temperature was maintained at 35 °C with a flow rate of 0.25 mL/min. A gradient with Mobile Phase A (HPLC-grade water) and Mobile phase B (95/5/5: Acetonitrile/2-propanol/HPLC grade water) was used to separate the components. The gradient (in % Mobile phase B) was as follows: 0–0.5 min: 90%; 0.5–15.5 min: 70%; 15.5–17 min: 70%; 17–17.1 min: 90%; 17.1–22 min: 90%. Injection volume was 5 µL and an external calibration curve was prepared to calculate the concentration of the analytes in the samples. The best fit curve was quadratic for all the carbohydrates.

HPLC with refractive index and ultraviolet detection was used to quantify glucose, pyruvate, acetate, ethanol, butyric acid, galactose, 1,2-propanediol, and lactic acid. 200 µL of metabolite sample was aliquoted into an HPLC vial with an insert (minimum 100 µL to avoid mis-injections) and ran without dilution. A set of independent control standards made in water were ran for each compound of interest (various concentrations were made to cover the expected range). The water was ultra-pure Millipore water from an in-house dispenser system (Purelab Ultra ELGA) and standards for each compound of interest (≥ 99% purity) were obtained from Sigma-Aldrich. Samples were vortexed, and analysis was performed using a 1200 HPLC system (Agilent) with Refractive Index and Ultraviolet detectors. A Bio-Rad Aminex HPX-87H column (300 × 7.8 mm, prepacked carbohydrate analysis column, hydrogen form, 9 µm particle size, 8% cross linkage, pH range 1–3) Part # 1,250,140 was used. The column temperature was maintained at 40 °C with a flow rate of 0.6 mL/min. Injection volume was 10 µL and external calibration check standards were prepared and used to confirm the validity of the calibration curves.

### RNA sequencing

Frozen cell pellets were sent to Genewiz (South Plainfield, NJ) for RNA isolation and sequencing. Cells were lysed using the Tissuelyser (Qiagen) and RNA was extracted using RNeasy Plus Mini Kit (Qiagen) following the manufacturer’s protocols. Ribosomal RNA was depleted using the Ribo-Zero Gold kit (Epicentre, Madison, WI, USA). RNA. Pooled libraries were sequenced on an Illumina HiSeq2500.

### Analysis of RNA-seq and differentially expressed genes

Paired-end RNA sequencing data were subsampled to 5 million reads per sample and interleaved into a single file using BBmerge version 1.1.3 with default settings within Geneious Prime version 2020.0.5 (Aukland, New Zealand). Paired reads were aligned to the Bi-26 genome using the Geneious alignment tool under the default settings. RNA-seq alignment summary statistics are in Supplemental Table 1.

We compared each carbohydrate source to no carbohydrate (focal carbohydrate as numerator, no carbohydrate as denominator). In contrast to previous studies, we did not use lactose as the base condition, as the global regulator effect of lactose could potentially obscure the differences in the signatures of the specific HMOs^26,28^, instead we used the no carbohydrate condition.

Differential expression analysis was performed using DESeq2 in Geneious^88^, and a gene was considered differentially expressed if the Benjamini–Hochberg adjusted *p* value < 0.05 and the absolute value of the log2 fold change > 1.5. Intersecting sets of differentially expressed genes were visualized with UpSet diagrams^89^ and log2 fold changes were visualized using the *pheatmap* package (version 1.0.12) in R (version 3.6.2). The reference genome for Bi-26 was prepared and sequenced according to previously published methods^24^.

## Supplementary information

Supplementary information

## Data Availability

*Bifidobacterium longum* subsp. *infantis* Bi-26 is safe deposited as ATCC SD-6720. The genome sequences for *B. longum* subsp. *infantis* Bi-26 and ATCC 15697 are available at the National Center for Biotechnology Information (NCBI) under Accession Numbers CP054425 and NC_011593. The expression data is available with the Gene Expression Omnibus Number XGSE151933.
