# Supplementary material for: Strain-specific strategies of 2′-fucosyllactose, 3-fucosyllactose, and difucosyllactose assimilation by Bifidobacterium longum subsp. infantis Bi-26 and ATCC 15697
Source: Sci Rep. 2020 Sep 28;10:15919. doi: 10.1038/s41598-020-72792-z (PMC7522266; doi:10.1038/s41598-020-72792-z)
Supplement: Supplementary file 1 — Supplementary information [file 41598_2020_72792_MOESM1_ESM.pdf]

SUPPLEMENTAL MATERIAL

Supplementary Figures 1-4 and Supplementary Tables 1-3 for manuscript:

Strain-Specific Strategies of 2′-Fucosyllactose, 3-Fucosyllactose, and Difucosyllactose Assimilation by *Bifidobacterium longum* subsp. *infantis* Bi-26 and ATCC 15697

Bryan E Zabel[1], Svetlana Gerdes[1], Kara C Evans[1], Derek Nedveck[1], Suzanne Koch Singles[2], Barbara Volk[2], Charles Budinoff[1]

[1]Genomics & Microbiome Science, DuPont Nutrition & Biosciences, Madison, WI, USA
[2]Advanced Analytical, DuPont Nutrition & Biosciences, Wilmington, DE, USA

Sample	Strain	Carbon Source	Control	RNA Concentration (ng/uL)	Number of Reads	subsampled read number	aligned reads	unaligned reads
Bi-26_T1_3FL_A	Bi-26	3FL	Bi-26_T1_NC	38.44	11377568	5000000	7740600	2259400
Bi-26_T1_3FL_B	Bi-26	3FL	Bi-26_T1_NC	23.57	12431150	5000000	8252389	1747611
Bi-26_T2_3FL_A	Bi-26	3FL	Bi-26_T2_NC	102.58	16230051	5000000	7770355	2229645
Bi-26_T2_3FL_B	Bi-26	3FL	Bi-26_T2_NC	17.76	11374518	5000000	8370200	1629800
Bi-26_T3_3FL_A	Bi-26	3FL	Bi-26_T2_NC	32.00	12184957	5000000	8116652	1883348
Bi-26_T3_3FL_B	Bi-26	3FL	Bi-26_T2_NC	92.97	12338227	5000000	8101526	1898474
Bi-26_T1_2FL_A	Bi-26	2'FL	Bi-26_T1_NC	13.07	14030434	5000000	9418727	581273
Bi-26_T1_2FL_B	Bi-26	2'FL	Bi-26_T1_NC	100.26	11550914	5000000	7741533	2258467
Bi-26_T2_2FL_A	Bi-26	2'FL	Bi-26_T2_NC	96.09	17227125	5000000	9326214	673786
Bi-26_T2_2FL_B	Bi-26	2'FL	Bi-26_T2_NC	10.56	13254370	5000000	8326915	1673085
Bi-26_T3_2FL_A	Bi-26	2'FL	Bi-26_T2_NC	82.94	13816879	5000000	8424651	1575349
Bi-26_T3_2FL_B	Bi-26	2'FL	Bi-26_T2_NC	80.58	14515764	5000000	9324944	675056
Bi-26_T1_DFL_A	Bi-26	DFL	Bi-26_T1_NC	9.58	16118479	5000000	9320234	679766
Bi-26_T1_DFL_B	Bi-26	DFL	Bi-26_T1_NC	9.87	13964078	5000000	9111017	888983
Bi-26_T2_DFL_A	Bi-26	DFL	Bi-26_T2_NC	77.02	11530159	5000000	7932688	2067312
Bi-26_T2_DFL_B	Bi-26	DFL	Bi-26_T2_NC	29.07	16465263	5000000	9444898	555102
Bi-26_T3_DFL_A	Bi-26	DFL	Bi-26_T2_NC	46.13	11644676	5000000	7727185	2272815
Bi-26_T3_DFL_B	Bi-26	DFL	Bi-26_T2_NC	37.26	12818912	5000000	8455592	1544408
Bi-26_T1_LAC_A	Bi-26	Lactose	Bi-26_T1_NC	10.59	11773090	5000000	7883415	2116585
Bi-26_T1_LAC_B	Bi-26	Lactose	Bi-26_T1_NC	70.95	13328524	5000000	9115290	884710
Bi-26_T2_LAC_A	Bi-26	Lactose	Bi-26_T2_NC	94.09	15192742	5000000	9844605	155395
Bi-26_T2_LAC_B	Bi-26	Lactose	Bi-26_T2_NC	14.83	11615198	5000000	8235262	1764738
Bi-26_T3_LAC_A	Bi-26	Lactose	Bi-26_T2_NC	29.68	10767323	5000000	7151390	2848610
Bi-26_T3_LAC_B	Bi-26	Lactose	Bi-26_T2_NC	99.03	12887162	5000000	8652072	1347928
Bi-26_T1_NC_A	Bi-26	No carbo	Control	2.93	11887067	5000000	7286597	2713403
Bi-26_T1_NC_B	Bi-26	No carbo	Control	2.93	11887067	5000000	8652010	1347990
Bi-26_T2_NC_A	Bi-26	No carbo	Control	8.13	10238382	5000000	7256215	2743785
Bi-26_T2_NC_B	Bi-26	No carbo	Control	50.80	14283924	5000000	8565450	1434550
Type_T2_3FL_A	ATCC 15697	3FL	Type_T2_NC	32.87	9655569	5000000	6717222	3282778
Type_T2_3FL_B	ATCC 15697	3FL	Type_T2_NC	90.89	13883429	5000000	7347634	2652366
Type_T2_2FL_A	ATCC 15697	2'FL	Type_T2_NC	25.85	11264287	5000000	7537164	2462836
Type_T2_2FL_B	ATCC 15697	2'FL	Type_T2_NC	30.97	13059233	5000000	7439038	2560962
Type_T2_DFL_A	ATCC 15697	DFL	Type_T2_NC	TOO LOW	12265619	5000000	7896696	2103304
Type_T2_DFL_B	ATCC 15697	DFL	Type_T2_NC	TOO LOW	12070465	5000000	6568626	3431374
Type_T2_LAC_A	ATCC 15697	Lactose	Type_T2_NC	40.65	8955163	5000000	7880264	2119736
Type_T2_LAC_B	ATCC 15697	Lactose	Type_T2_NC	75.18	11984846	5000000	7488110	2511890
Type_T2_GLU_A	ATCC 15697	Glucose	Type_T2_NC	5.87	12054331	5000000	7522120	2477880
Type_T2_GLU_B	ATCC 15697	Glucose	Type_T2_NC	8.82	17937551	5000000	7623448	2376552
Type_T2_FUC_A	ATCC 15697	Fucose	Type_T2_NC	3.87	10070561	5000000	7725412	2274588
Type_T2_FUC_B	ATCC 15697	Fucose	Type_T2_NC	2.55	11989395	5000000	7112358	2887642
Type_T2_NC_A	ATCC 15697	No carbo	Control	3.42	12103669	5000000	7726618	2273382
Type_T2_NC_B	ATCC 15697	No carbo	Control	TOO LOW	11001749	5000000	7936382	2063618

Supplemental Table 1: RNA-seq quality statistics. Quality data for RNA concentration (qubit), number of reads generated, number of reads sampled and aligned are shown for all the samples. The controls that were used for comparison for RNA-seq is also listed.

Locus ID	Abbreviation	Protein function
n/a	Aceto-hydroxybut	2-aceto-2-hydroxybutanoate
Blon_1961	ADH	Aldehyde dehydrogenase (EC 1.2.1.3)
Blon_2241	ALH	Alcohol dehydrogenase (EC 1.1.1.1)
Blon_2239	ALDC	Alpha-acetolactate decarboxylase (EC 4.1.1.5)
Blon_0399	ALS_a	Acetolactate synthase large subunit (EC 2.2.1.6)
Blon_0398	ALS_b	Acetolactate synthase small subunit (EC 2.2.1.6)
Blon_1731	askA	Acetate kinase (EC 2.7.2.1)
Blon_1836	Eno	Enolase (EC 4.2.1.11)
Blon_0538	GalE	UDP-glucose-4-epimerase (EC 5.1.3.2)
Blon_2062	GalK	Galactokinase (EC 2.7.1.6)
Blon_2063	GalT	galT, Galactose-1-phosphate uridylyltransferase (EC 2.7.7.10)
Blon_0565	GLK	Glucokinase (EC 2.7.1.2)
Blon_0417	GPI	Glucose-6-phosphate isomerase (EC 5.3.1.9)
Blon_0131	ilvC	Ketol-acid reductoisomerase (NADP(+)) (EC 1.1.1.86)
Blon_0136	ilvC	Ketol-acid reductoisomerase (NADP(+)) (EC 1.1.1.86)
Blon_2134	ilvD	Dihydroxy-acid dehydratase (EC 4.2.1.9)
Blon_0540	LAR	Lactaldehyde reductase (EC 1.1.1.77) -- to 1,2PD
Blon_0840	LDH	L-lactate dehydrogenase (EC 1.1.1.27)
Blon_1090	LDH	L-lactate dehydrogenase (EC 1.1.1.27)
Blon_0059	PEPC	Phosphoenolpyruvate carboxylase (EC 4.1.1.31)
Blon_1715	PFL	Pyruvate formate-lyase (EC 2.3.1.54)
Blon_1714	PFL_act	Pyruvate formate-lyase activating enzyme (EC 1.97.1.4)
Blon_2184	PGM	Phosphoglucomutase (EC 5.4.2.2)
Blon_1730	PTA	Phosphate acetyltransferase (EC 2.3.1.8)
Blon_1745	PyK	Pyruvate kinase (EC 2.7.1.40)
Blon_1095	TAL	Transaldolase (EC 2.2.1.2)
Blon_1096	TKL	Transketolase (EC 2.2.1.1)
Blon_1722	XFP	Xylulose-5P/Fructose-6P phosphoketolase (EC 4.1.2.9; EC 4.1.2.22)

Supplemental Table 2. Gene names and protein functions for abbreviations used in Figure 2.

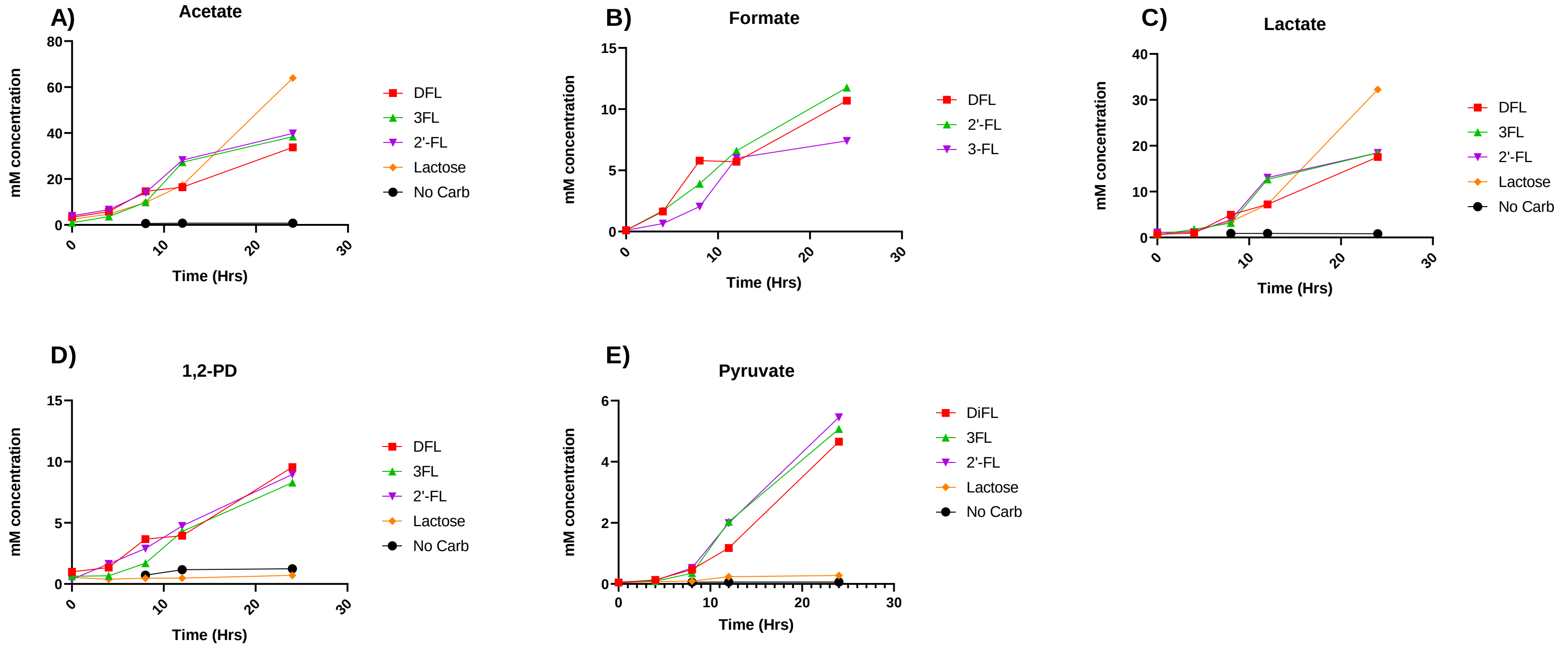

Supplemental Figure 1: Metabolite data for Bi-26 growth on FLs. Acetate (A), Formate (B), Lactate (C), 1,2-PD (D), and pyruvate (E) were measured over the 24-hour growth period. Two replicates of each timepoint were taken at T1-T4.

A)

Acetate:Lactate	3-FL	2'-FL	diFL	Lactose
ATCC15697	4.75:1	4.37:1	9.33:1	1.75:1
Bi-26	2.08:1	2.15:1	1.92:1	1.98:1

B)

Lactate:Formate	3-FL	2'-FL	diFL
ATCC15697	0.52:1	0.38:1	0.33:1
Bi-26	2.49:1	1.57:1	1.64:1

Supplemental Table 3: Ratios of acetate to lactate (A) and lactate to formate (B) for Bi-26 and ATCC15697 for the different carbon sources.

A)

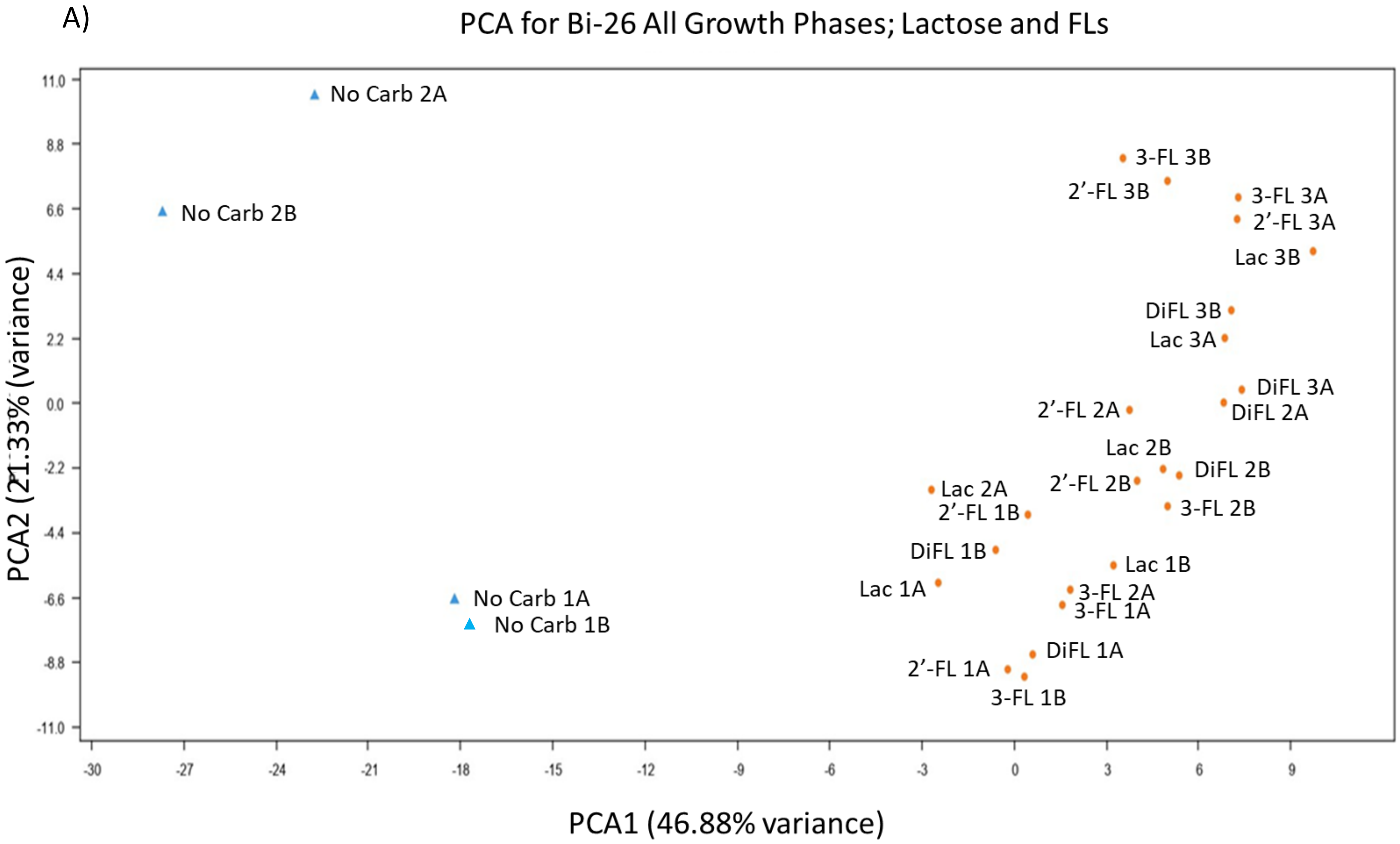
PCA for Bi-26 All Growth Phases; Lactose and FLs
PCA2 (21.33% (variance)
PCA1 (46.88% variance)
11.0
8.8
6.6
4.4
2.2
0.0
-2.2
-4.4
-6.6
-8.8
-11.0
-30
-27
-24
-21
-18
-15
-12
-9
-6
-3
0
3
6
9
No Carb 2A
No Carb 2B
No Carb 1A
No Carb 1B
3-FL 3B
2'-FL 3B
3-FL 3A
2'-FL 3A
Lac 3B
DiFL 3B
Lac 3A
DiFL 3A
2'-FL 2A
DiFL 2A
Lac 2B
DiFL 2B
2'-FL 2B
Lac 2A
2'-FL 1B
3-FL 2B
DiFL 1B
Lac 1B
Lac 1A
3-FL 2A
3-FL 1A
DiFL 1A
2'-FL 1A
3-FL 1B

B)

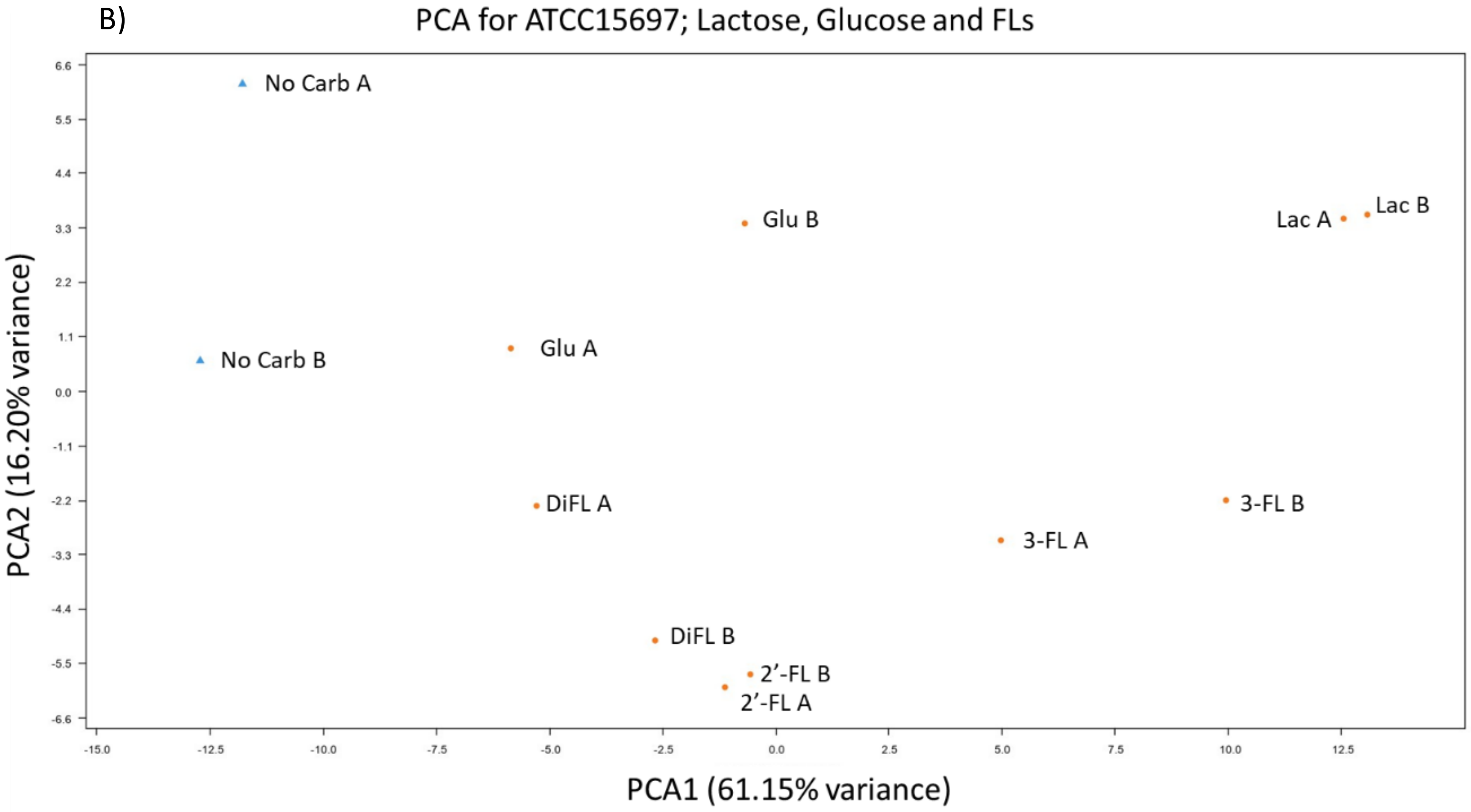

Supplemental Figure 2. PCA plot of Bi-26 (A) and ATCC 15697 (B) transcriptomes for lactose, glucose, FLs and the no carbohydrate control to show their likeness. The Bi-26 samples at time points T1, T2 and T3 are signified by 1, 2, and 3. Biological replicates are signified by the letters A and B. A single time point for each carbon source was analyzed for ATCC15697, corresponding to T2.

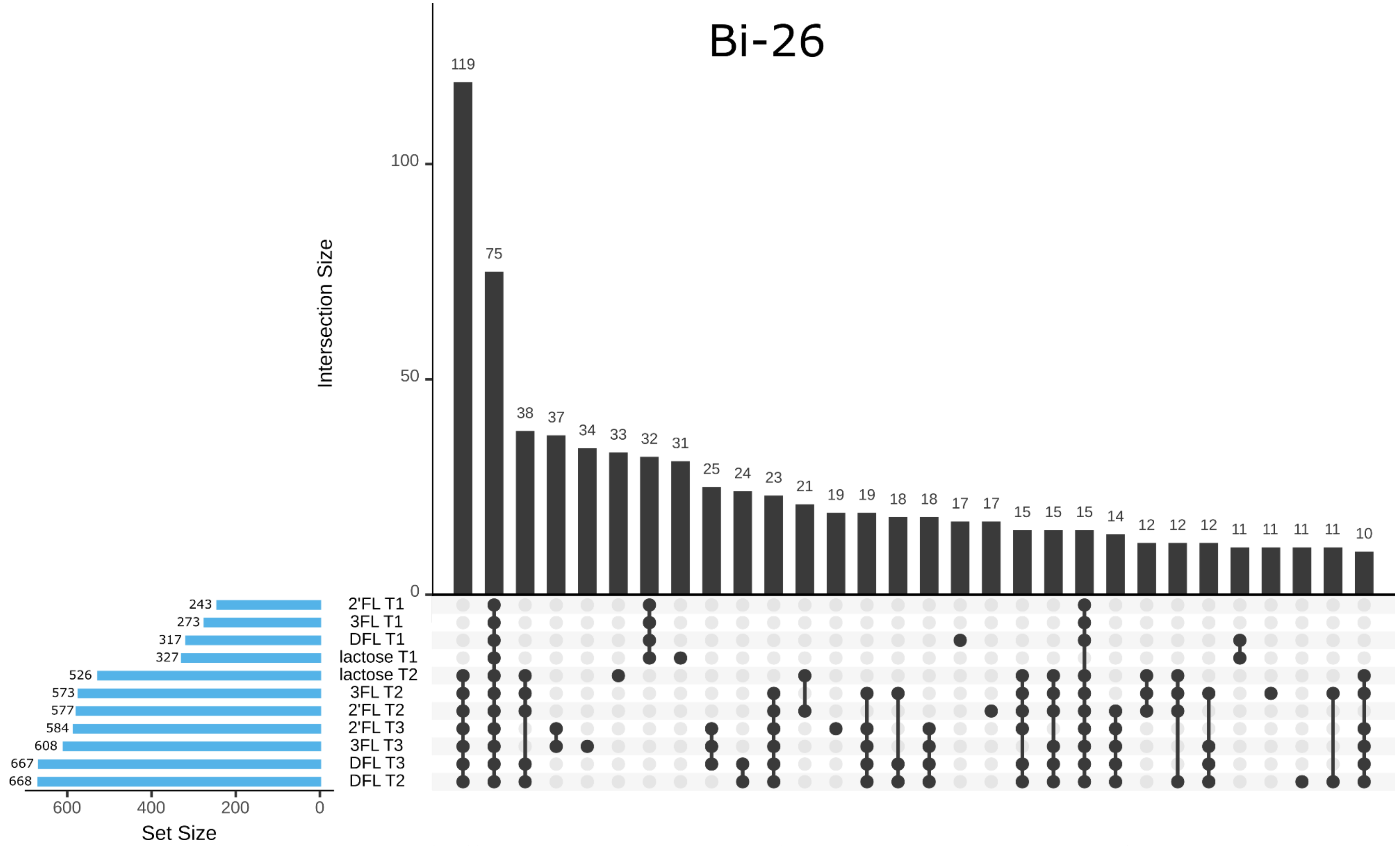

Supplemental Figure 3. Upset diagram of differential gene counts (DEGs) for all conditions that were tested for Bi-26. Total number of DEGs is shown on the left (blue bars) for each of the conditions while the number of shared genes per condition (shown by the connected dots), is located on the black bars.

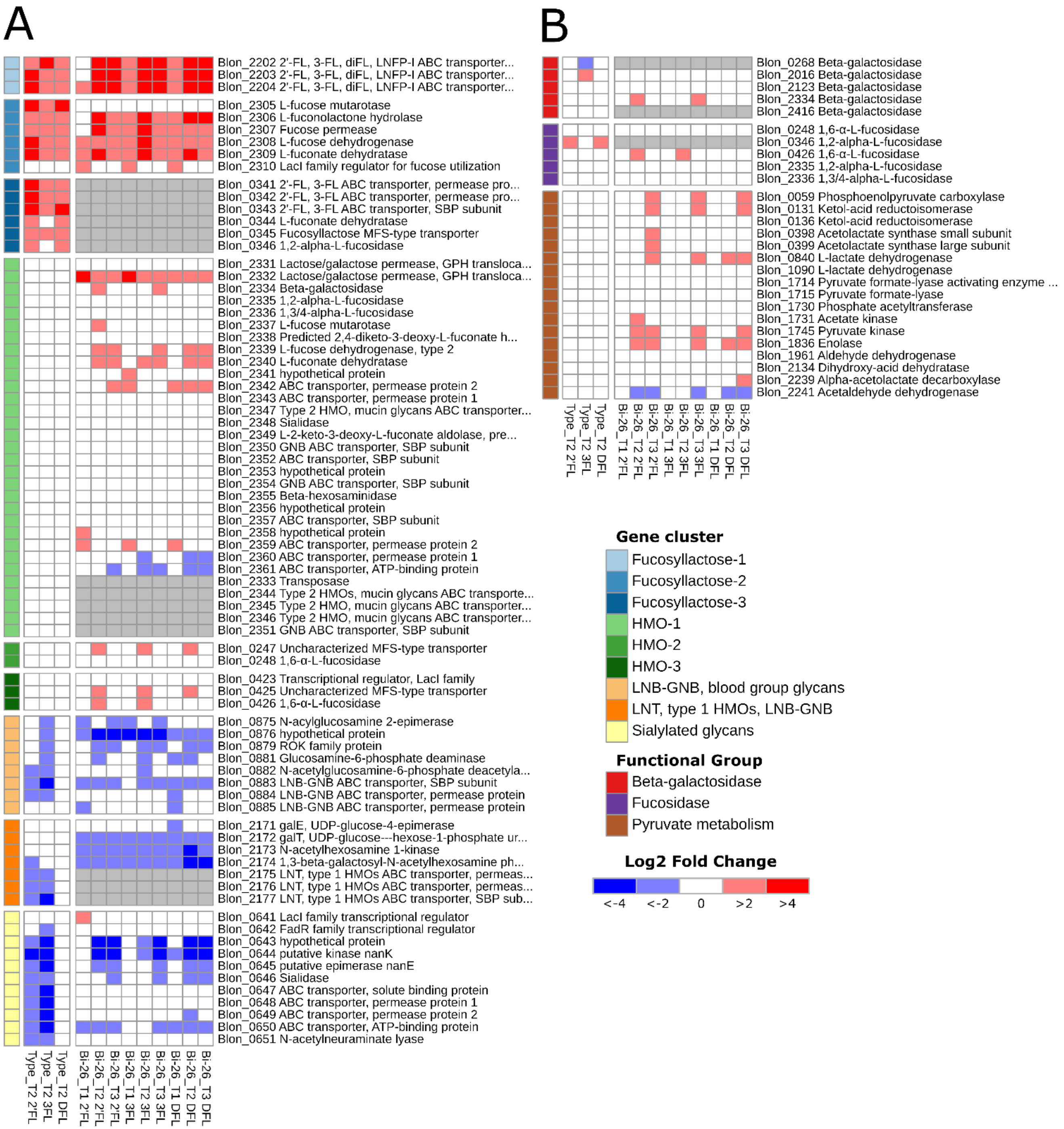

A
Blon_2202 2'-FL, 3-FL, diFL, LNFP-I ABC transporter...
Blon_2203 2'-FL, 3-FL, diFL, LNFP-I ABC transporter...
Blon_2204 2'-FL, 3-FL, diFL, LNFP-I ABC transporter...
Blon_2305 L-fucose mutarotase
Blon_2306 L-fuconolactone hydrolase
Blon_2307 Fucose permease
Blon_2308 L-fucose dehydrogenase
Blon_2309 L-fuconate dehydratase
Blon_2310 LacI family regulator for fucose utilization
Blon_0341 2'-FL, 3-FL ABC transporter, permease pro...
Blon_0342 2'-FL, 3-FL ABC transporter, permease pro...
Blon_0343 2'-FL, 3-FL ABC transporter, SBP subunit
Blon_0344 L-fuconate dehydratase
Blon_0345 Fucosyllactose MFS-type transporter
Blon_0346 1,2-alpha-L-fucosidase
Blon_2331 Lactose/galactose permease, GPH transloca...
Blon_2332 Lactose/galactose permease, GPH transloca...
Blon_2334 Beta-galactosidase
Blon_2335 1,2-alpha-L-fucosidase
Blon_2336 1,3/4-alpha-L-fucosidase
Blon_2337 L-fucose mutarotase
Blon_2338 Predicted 2,4-diketo-3-deoxy-L-fuconate h...
Blon_2339 L-fucose dehydrogenase, type 2
Blon_2340 L-fuconate dehydratase
Blon_2341 hypothetical protein
Blon_2342 ABC transporter, permease protein 2
Blon_2343 ABC transporter, permease protein 1
Blon_2347 Type 2 HMO, mucin glycans ABC transporter...
Blon_2348 Sialidase
Blon_2349 L-2-keto-3-deoxy-L-fuconate aldolase, pre...
Blon_2350 GNB ABC transporter, SBP subunit
Blon_2352 ABC transporter, SBP subunit
Blon_2353 hypothetical protein
Blon_2354 GNB ABC transporter, SBP subunit
Blon_2355 Beta-hexosaminidase
Blon_2356 hypothetical protein
Blon_2357 ABC transporter, SBP subunit
Blon_2358 hypothetical protein
Blon_2359 ABC transporter, permease protein 2
Blon_2360 ABC transporter, permease protein 1
Blon_2361 ABC transporter, ATP-binding protein
Blon_2333 Transposase
Blon_2344 Type 2 HMOs, mucin glycans ABC transporte...
Blon_2345 Type 2 HMO, mucin glycans ABC transporter...
Blon_2346 Type 2 HMO, mucin glycans ABC transporter...
Blon_2351 GNB ABC transporter, SBP subunit
Blon_0247 Uncharacterized MFS-type transporter
Blon_0248 1,6-α-L-fucosidase
Blon_0423 Transcriptional regulator, LacI family
Blon_0425 Uncharacterized MFS-type transporter
Blon_0426 1,6-α-L-fucosidase
Blon_0875 N-acylglucosamine 2-epimerase
Blon_0876 hypothetical protein
Blon_0879 ROK family protein
Blon_0881 Glucosamine-6-phosphate deaminase
Blon_0882 N-acetylglucosamine-6-phosphate deacetyla...
Blon_0883 LNB-GNB ABC transporter, SBP subunit
Blon_0884 LNB-GNB ABC transporter, permease protein
Blon_0885 LNB-GNB ABC transporter, permease protein
Blon_2171 galE, UDP-glucose-4-epimerase
Blon_2172 galT, UDP-glucose---hexose-1-phosphate ur...
Blon_2173 N-acetylhexosamine 1-kinase
Blon_2174 1,3-beta-galactosyl-N-acetylhexosamine ph...
Blon_2175 LNT, type 1 HMOs ABC transporter, permeas...
Blon_2176 LNT, type 1 HMOs ABC transporter, permeas...
Blon_2177 LNT, type 1 HMOs ABC transporter, SBP sub...
Blon_0641 LacI family transcriptional regulator
Blon_0642 FadR family transcriptional regulator
Blon_0643 hypothetical protein
Blon_0644 putative kinase nanK
Blon_0645 putative epimerase nanE
Blon_0646 Sialidase
Blon_0647 ABC transporter, solute binding protein
Blon_0648 ABC transporter, permease protein 1
Blon_0649 ABC transporter, permease protein 2
Blon_0650 ABC transporter, ATP-binding protein
Blon_0651 N-acetylneuraminate lyase
Type_T2 2'FL
Type_T2 3FL
Type_T2 DFL
Bi-26_T1 2'FL
Bi-26_T2 2'FL
Bi-26_T3 2'FL
Bi-26_T1 3FL
Bi-26_T2 3FL
Bi-26_T3 3FL
Bi-26_T1 DFL
Bi-26_T2 DFL
Bi-26_T3 DFL
B
Blon_0268 Beta-galactosidase
Blon_2016 Beta-galactosidase
Blon_2123 Beta-galactosidase
Blon_2334 Beta-galactosidase
Blon_2416 Beta-galactosidase
Blon_0248 1,6-α-L-fucosidase
Blon_0346 1,2-alpha-L-fucosidase
Blon_0426 1,6-α-L-fucosidase
Blon_2335 1,2-alpha-L-fucosidase
Blon_2336 1,3/4-alpha-L-fucosidase
Blon_0059 Phosphoenolpyruvate carboxylase
Blon_0131 Ketol-acid reductoisomerase
Blon_0136 Ketol-acid reductoisomerase
Blon_0398 Acetolactate synthase small subunit
Blon_0399 Acetolactate synthase large subunit
Blon_0840 L-lactate dehydrogenase
Blon_1090 L-lactate dehydrogenase
Blon_1714 Pyruvate formate-lyase activating enzyme ...
Blon_1715 Pyruvate formate-lyase
Blon_1730 Phosphate acetyltransferase
Blon_1731 Acetate kinase
Blon_1745 Pyruvate kinase
Blon_1836 Enolase
Blon_1961 Aldehyde dehydrogenase
Blon_2134 Dihydroxy-acid dehydratase
Blon_2239 Alpha-acetolactate decarboxylase
Blon_2241 Acetaldehyde dehydrogenase
Type_T2 2'FL
Type_T2 3FL
Type_T2 DFL
Bi-26_T1 2'FL
Bi-26_T2 2'FL
Bi-26_T3 2'FL
Bi-26_T1 3FL
Bi-26_T2 3FL
Bi-26_T3 3FL
Bi-26_T1 DFL
Bi-26_T2 DFL
Bi-26_T3 DFL
Gene cluster
Fucosyllactose-1
Fucosyllactose-2
Fucosyllactose-3
HMO-1
HMO-2
HMO-3
LNB-GNB, blood group glycans
LNT, type 1 HMOs, LNB-GNB
Sialylated glycans
Functional Group
Beta-galactosidase
Fucosidase
Pyruvate metabolism
Log2 Fold Change
<-4
<-2
0
>2
>4

Supplemental Figure 4. Differential expression of the HMO utilization genes within the global transcriptomes of ATCC 15697 and Bi-26 (additional timepoint shown) strains grown on 2’FL, 3FL, or DFL shown as log2-fold change. Transcriptomes of cultures grown in mBasal medium with no carbon source added were used as a reference. A fold change ≥ 1.5-fold and Benjamini–Hochberg adjusted p-value < 0.05 were considered for statistical significance. Orthologous genes between the Bi-26 and ATCC 15697 are shown on the same rows and are identified by Blon IDs. **Panel A**: Differential expression of the gene loci implicated in recognition, internalization, and intracellular hydrolysis of fucosyllactose and several other HMO species in *B. longum* subsp. *infantis*. Genes are shown in the order on the chromosome. General HMO specificity of each locus is color-coded on the left. In gray are the orthologs of ATCC 15697 genes missing from the genome of Bi-26. Most of the missing genes are transporter systems for various HMOs, which underscores the Bi-26 apparent preference for 2’FL, 3FL, and DFL utilization. Upregulation of the L-fucose utilization genes in the HMO-1 cluster in Bi-26, but not in ATCC 15697 is another indication of a regulatory adaptation to preferential utilization of small fucosylated HMOs by Bi-26. **Panel B:** Differential expression of metabolic genes involved in utilization of 2’FL, 3FL, DFL and their constituent monosaccharides. See Supplemental Table 2 for explanation of abbreviations.
